# Case presentation of patients hospitalised with mpox (subclade Ib/2023sh) including children, adolescents, and adults in South Kivu, Democratic Republic of the Congo: an observational cohort study

**DOI:** 10.1016/S1473-3099(26)00051-4

**Published:** 2026-06

**Authors:** Luis Flores Girón, Gustavo Sganzerla Martinez, Baganda Ntahuma Daniel, Alfred Kesheni Bisimwa, Nkonzi Pacific, Georges Assumani Martin, Jean Christian Amini Kabwana, Anuj Kumar, Ali Toloue Ostadgavahi, Mansi Dutt, Kawaya Lusante Bénite, Mangura Hamuli Damien, Christian Gortázar, Bahaa Abu-Raya, Alyson A Kelvin, Kaleme Kiswele Prince, David J Kelvin

**Affiliations:** a Lwiro Primate Rehabilitation Center, Lwiro, Democratic Republic of the Congo; b Centre de Recherche en Sciences Naturelles de Lwiro, Lwiro, Democratic Republic of the Congo; c One Health Conservation Initiative, Katana, Democratic Republic of the Congo; d Department of Immunology, Shantou University Medical College, Shantou, China; e Department of Microbiology and Immunology, Faculty of Medicine, Dalhousie University, Halifax, NS, Canada; f Department of Pediatrics, Faculty of Medicine, Dalhousie University, Halifax, NS, Canada; g SaBio IREC Universidad de Castilla La Mancha, Ciudad Real, Spain; h Canadian Center for Vaccinology, Dalhousie University, Izaak Walton Killam (IWK) Health Centre, and the Nova Scotia Health Authority, Halifax, NS, Canada; i Faculty of Veterinary Medicine, University of Calgary, Calgary, AL, Canada; j Institut Superieur des Techniques Medicales de Bukavu, Bukavu, Democratic Republic of the Congo; k Department of Pediatrics, IWK Health Center, Canadian Center for Vaccinology, Halifax, NS, Canada; l Joint PhD Program, Department of Biomedical Sciences, University of Sassari, Sassari, Italy; m Université du Cinquantenaire, Lwiro, Democratic Republic of the Congo

## Abstract

**Background:**

Mpox is a public health concern in eastern DR Congo. It continues to cause substantial numbers of hospital admissions, with changing demographics including children and adolescents, requiring comprehensive clinical and epidemiological investigation. In this study, we aim to describe the clinical characteristics of hospitalised participants infected with monkeypox virus (MPXV) subclade Ib/2023sh in the Kabare Territory in South Kivu, DR Congo.

**Methods:**

This observational cohort study included patients admitted with suspected mpox to the reference centre of mpox treatment at Lwiro Hospital, South Kivu, DR Congo. Eligible participants must have had, at the time of inclusion, skin lesions compatible with the infection. Individuals who did not present lesions compatible with MPXV infection were also eligible if they had at least one of the following symptoms: fever, cervical lymphadenopathy, or pharyngitis, provided they had been in contact with someone with suspected mpox within the last 21 days. Data from hospital records and standardised clinical forms captured demographics, presenting symptoms and signs, outcomes, and general clinical characteristics. Descriptive analyses and statistics summarised the clinical and epidemiological profiles of participants with molecular confirmation of MPXV subclade Ib/2023sh.

**Findings:**

Between Aug 3, 2024, and Feb 8, 2025, MPXV subclade Ib/2023sh was detected in 494 (77%) of 643 participants with a median age of 9 years (IQR 2–24). Participants who were positive for MPXV subclade Ib/2023sh infection were more often female (290 [59%]) and were generally older (median 16 years [4–25]) than male participants (204 [41%]; median age 4 years [1–14]). 300 (61%) of 494 participants were aged 15 years or younger. Fever (444 [90%]), skin lesions or rash (391 [79%]), and dysphagia (279 [56%]) were the most prevalent symptoms. Children aged 0–5 years had a higher frequency of lesions on the head (84 [41%] of 203), face (67 [33%]), neck (23 [11%]), back (27 [13%]), arm (35 [17%]), palm of hand (35 [17%]), chest (46 [23%]), posterior aspect of thighs (40 [20%]), legs (25 [12%]), dorsal foot (45 [22%]), and oral cavity (37 [18%]). 117 (24%) participants had lesions in the oral cavity. Oral cavity and oropharynx swabs were able to detect MPXV subclade Ib/2023sh in the absence of assayable skin lesions.

**Interpretation:**

The high proportion of children and adolescents (aged ≤15 years) differentiates our cohort from other clinical descriptions of the novel MPXV subclade Ib/2023sh. Given that, we hypothesise a demographic shift in the target population that contributes to the community spread of mpox in the South Kivu region of DR Congo. Targeted public health measures should consider ways to reduce transmission among children and adolescents.

**Funding:**

Canadian Institutes of Health Research (CIHR), Canadian Foundation for Innovation, Research Nova Scotia, Dalhousie Medical Foundation, Moderna, Li-Ka Shing Foundation, European & Developing Countries Clinical Trials Partnership (EDCTP).

**Translations:**

For the French, Swahili and Mashi translations of the abstract see Supplementary Materials section.

## Introduction

Mpox is a viral disease caused by *Orthopoxvirus monkeypox* (MPXV). MPXV is phylogenetically divided into two distinct clades (I and II).^1^ Human mpox was first documented in the 1970s in DR Congo. Initially, mpox outbreaks were predominantly characterised by small, self-limited clusters of cases, with most infections linked to zoonotic transmission through direct contact with wildlife.^2^, ^3^, ^4^ In the 21st century, the number of mpox cases has increased in endemic and non-endemic areas with travel-associated spread. In Africa alone, the rising number of cases, especially after 2022, is cause for increased international concern.^5^ Subclades Ib and IIb of MPXV have shown a high capacity for sustained human-to-human transmission. In 2022, a multicountry outbreak prompted WHO to declare a public health emergency of international concern due to unprecedented global spread of MPXV subclade IIb/2017sh.^6^, ^7^, ^8^, ^9^, ^10^ As of Nov 2, 2025, there were more than 165 000 confirmed cases and 441 deaths in 140 countries worldwide.^11^ In late 2023, the novel MPXV subclade Ib/2023sh was identified in eastern DR Congo (Kamituga, South Kivu).^12^, ^13^ This virus had a unique set of genomic differences yielding a separate subclade Ib in phylogenetic analyses.^14^ Initial transmission chains in the MPXV subclade Ib/2023sh outbreak were linked to professional sex workers, with viral spread occurring primarily through close contact during sexual activities, although non-sexual transmission was also documented.^15^ As the outbreak progressed, subsequent transmission extended well beyond sexual networks, resulting in intrafamily and interfamily spread within affected communities.^15^ Sustained human-to-human transmission within DR Congo and neighbouring countries was documented, with community spread and travel-related cases reported internationally.^16^ WHO declared a second public health emergency of international concern in August, 2024.

**Research in context**

**Evidence before this study**

We searched PubMed for cohort studies published from database inception to Sept 26, 2025, using the terms “monkeypox”, OR “mpox”, OR “monkey pox”, OR “monkeypox virus”, OR “MPXV”, OR “monkey pox virus” to find information on the age distribution of cases. We identified 40 research articles with demographic data available to be extracted. The decades after the first reported human case of mpox were highlighted by zoonotic infections with limited secondary infections. Nine studies from 1970–99 were found in which children and adolescents comprised 372 (81%) of the 457 identified participants. We found 18 studies published between 2000 and 2020 in which children and adolescents comprised 610 (58%) of the 1046 identified participants. Most cases were associated with zoonotic infections. The outbreaks found in the decade of 2020s were dominated by the 2022 *Orthopoxvirus monkeypox* (MPXV) subclade IIb/2017sh multicountry outbreak. In the six studies with participant data available from this outbreak, only a small number of the 4348 identified participants were children. The sustained human-to-human transmission aspect of this outbreak was highlighted by close-contact transmission involving sexual activities. In 2023, the introduction of subclade Ib/2023sh featured sustained human-to-human transmission of mpox clade I viruses. Four cohort studies containing age demographics were found where 332 of the 1458 participants across the four studies were children. There are limited reports of detailed clinical symptoms of MPXV subclade Ib/2023sh infections in children and their possible involvement in sustained human-to-human transmission of mpox remains unclear.

**Added value of this study**

Our study describes a high proportion of paediatric hospitalisations (60% of the confirmed cases) in an outbreak of MPXV subclade Ib/2023sh in a hospital located in South Kivu, DR Congo. Children and adolescents (≤15 years) comprised 300 (61%) of the 494 study participants who were PCR positive. The households of study participants contain a median number of six individuals where on average, four members are aged younger than 15. Our clinical description of cases includes children aged 5 years or younger having substantially more lesions on the head, face, neck, back of arms, palm of hand, posterior aspect of thighs, legs, dorsal foot, and in the oral cavity, while adults aged 21 years or older had more lesions on the genitalia. The analysis of molecular diagnostics suggests detectable MPXV subclade Ib/2023sh in the oral cavity before the development of skin lesions.

**Implications of all the available evidence**

This study shows a high proportion of children and adolescents infected during an outbreak of MPXV subclade Ib/2023sh. Our results have important clinical applications that can inform clinical decision making of relevant signs, symptoms, and lesion distribution of MPXV subclade Ib/2023sh infections, especially in children and adolescents. We also identified a high positivity rate of detecting MPXV subclade Ib/2023sh in oral cavity samples which might arise before the development of a skin rash or lesions, making oral cavity swabbing a potentially effective alternative mpox diagnostic method in the absence of easily assayable skin lesions.

Children and adolescents aged 15 years or younger were not the predominant demographic in clinical cohorts of MPXV subclade Ib/2023sh, limiting available clinical data in paediatric populations.^12^, ^15^, ^17^, ^18^ This study aims to address knowledge gaps by describing the clinical characteristics of mpox (MPXV subclade Ib/2023sh) in a predominantly paediatric hospitalised cohort in Lwiro Hospital located in the Kabare Territory, Miti-Murhesa health area, South Kivu, DR Congo.

## Methods

### Study design and participants

This observational cohort study included participants who were admitted to the Centre de Recherche en Sciences Naturelles (CRSN) Lwiro Hospital (Kabare Territory, Miti-Murhesa health area, South Kivu) between Aug 3, 2024 and Feb, 8, 2025. Lwiro Hospital belongs to the Centre de Recherche en Sciences Naturelles and is included in the network of hospitals under the direction of the Division Provincial de Santé de Sud-Kivu. The inclusion criteria used in this study comprised individuals of any age in the Miti-Murhesa health zone, where the mpox epidemic was endemic and showed strong community transmission. Participants were required to present, at the time of inclusion, skin lesions compatible with the infection. Individuals who did not present lesions compatible with mpox infection were also eligible if they had at least one of the following symptoms: fever, cervical lymphadenopathy, or pharyngitis, provided they had been in contact with someone with suspected mpox within the last 21 days. The inclusion criteria adopted in our research were based on the recommendations of the Miti-Murhesa health zone in the South Kivu province for admission to mpox treatment centres, with modifications to allow us to recruit relatives of participants who had close contact with individuals with suspected mpox. Ethical approval for conducting this study was approved by the Comite Institutionnel D'Ethique de la Sante (ISTM-BKV/CRPS/CIES/MLM/007/2024 and ISTM-BKV/CRPS/CIES/ML/011/2024) and Izaak Walton Killam (IWK) Review Ethics Board (1030215). Memorandum of understanding, material and data sharing agreements, and import and export permits were obtained for relevant partners (Centre de Recherche en Sciences Naturelles–Lwiro, IWK Health Centre, and Dalhousie University) for this study. All study participants were introduced to the study and given the option to participate. Consenting participants provided written informed consent or, in the case that the participant was younger than 18 years, parental or legal guardian consent was obtained. For participants aged 12–17 years, age-specific verbal assent was also obtained in accordance with the law of DR Congo. All patients who were hospitalised at the mpox treatment centre of Lwiro Hospital, designated as a *centre de traitement officiel de Mpox* by the Division Provinciale de la Santé du Sud-Kivu, received medical treatment with the necessary hygiene measures according to established protocols and medical judgement, and were provided with accommodation, a bed, and food until full recovery. Given the public health emergency in South Kivu during the study period, participant enrolment was guided not only by predefined criteria but also by the judgement of the attending clinicians. Hospitalisation and care were provided regardless of participation status. All participant data were de-identified before analysis. The study team, including clinicians and members of the writing group, did not have access to any personally identifiable information. Confidential documentation is secured in a locked vault in a locked room. Keys and data are only accessible to individuals who have ethical clearance to examine and analyse the data.

### Procedures

Participants were evaluated for clinical symptoms (eg, fever and level of pain) and physical signs, including the presence and anatomical distribution of skin lesions. Swabs from the oral cavity or oropharynx were collected (mouth, sublingual, oropharynx, or pharynx) as well as skin swabs (crusts, vesicles, pustules, macules, or papules in different non-oral anatomical regions) for subsequent analysis. Swabs were collected at least once from skin samples that could be opened to collect the internal content that were present. Otherwise, only a swab from the oral cavity or oropharynx was obtained. The collected swabs were used for the molecular detection of MPXV subclade Ib/2023sh through amplification refractory mutation system-based quantitative real-time PCR using the MPXV subclade Ib/2023sh-specific primers proposed and validated by Xu and colleagues.^19^ The PCR assay was validated with internal controls and cross-referenced with samples from the early Kamituga outbreak,^8^ where the samples were sequenced to validate the PCR system. The molecular facility where the PCRs were conducted was operating only at research capacity and could not provide molecular diagnostics for clinical use. Results from the study were communicated to the clinicians only in general terms or in figures designed for presentation or communication. No results were communicated to patients. Additional documentation on the PCR reactions is included in appendix 4 (p 6). Skin and oral cavity and oropharyngeal swabs were used for DNA extraction and further DNA metagenomics sequencing using the Oxford Nanopore Rapid Sequencing platform. Additional details on the sequencing methods are included in appendix 4 (p 7).

### Outcomes

Clinical symptoms and physical signs, including the presence and anatomical distribution of skin lesions, were recorded and counted. Laboratory outcomes include MPXV subclade Ib/2023sh genotyping and viral load measurements from oral cavity, oropharyngeal, or skin lesion swabs. When feasible, skin lesion material was collected at the visible, pustule, or scab stage for additional testing. Disease severity markers, including length of hospitalisation and mortality, were also recorded. Additional details on the outcome variables and criteria used by hospital staff are included in appendix 4 (p 4). The outcomes were analysed in all participants regardless of their mpox PCR results.

The collected data was explored in Python (version 3.10). Data was tested for normality using the Shapiro–Wilk test. Continuous data were compared using *t* test, Mann–Whitney *U* test, ANOVA, and Kruskal–Wallis test, depending on the number of groups compared and data normality. Association between categorical variables was assessed using the χ^2^ test of independence. p values less than 0·05 were considered as statistically significant. 95% CIs were calculated for categorical variables expressed as proportions and for continuous variables. Missing values for clinical variables were minimal (<1%) and were excluded from the relevant analysis. No imputation was performed, and analyses were conducted on available data only. The statistical analysis plan is included in appendix 4 (p 5). To calculate a combined maximum-likelihood phylogeny tree of previously reported clade Ia (39) and Ib (536) and ten Central South Kivu sequences obtained in this study, we used the IQ-TREE multicore package (version 2.2.6) embedded in the Squirrel pipeline.

### Role of the funding source

The funders of the study had no role in study design, data collection, data analysis, data interpretation, or writing of the report.

## Results

Between Aug 3, 2024, and Feb 8, 2025, 929 participants with suspected mpox infection at Lwiro Hospital were recruited and enrolled to this study. Participants who consented to participate represented more than 90% of individuals approached for enrolment into the study. Due to armed conflict in the eastern region of DR Congo during the study period, only 643 participants had samples collected for the molecular detection of MPXV subclade Ib/2023sh. Of these, 494 (77%) of 643 samples were positive for viral MPXV DNA. It took a median of 4 days (IQR 3–7) from the first onset of symptoms until participants presented to hospital. The median age of the participants was 9 years (2–24). 300 (61%) of 494 participants were aged 15 years or younger. Participants who were positive for MPXV DNA were more often female (290 [59%]) and were generally older (median 16 years [4–25]) than male participants (204 [41%]; median age 4 years [1–14]). 230 participants reported previous contact with an individual with suspected mpox and were therefore eligible for recruitment in the absence of skin lesions. Further demographics and epidemiological details of participants who were PCR positive are summarised in the table. Rapid diagnostic testing for malaria was conducted in 237 participants with 21 being positive. We include the demographics and epidemiological details of all 929 recruited participants in appendix 4 (pp 2–3).

**Table:** Baseline characteristics of the included population

		**Female (n=290)**	**Male (n=204)**	**Total (n=494)**
**Baseline characteristics**				
Age, years		16 (4–25)	4 (1–14)	9 (2–24)
Age at admission				
	<1 year	35 (12%)	56 (27%)	91 (18%)
	1–5 years	56 (20%)	56 (27%)	112 (23%)
	6–10 years	33 (11%)	26 (13%)	59 (12%)
	11–15 years	20 (7%)	18 (9%)	38 (8%)
	16–20 years	33 (11%)	14 (7%)	47 (10%)
	≥21 years	113 (4%)	34 (17%)	147 (30%)
Median household size		6 (5–8)	7 (5–9)	6 (5–8)
Median number of people aged ≤15 years in the same household		4 (3–6)	4 (3–6)	4 (3–6)
Median number of people aged >15 years in the same household		2 (2–2)	2 (2–2)	2 (2–2)
**Risk factors and clinical examination**				
Contact with suspect mpox case		148 (51%)	82 (40%)	230 (47%)
Pregnancy		13 (4%)	..	13 (3%)
Active smoking		0	4 (2%)	4 (1%)
Previous tuberculosis		0	1 (<1%)	1 (<1%)
HIV		2 (1%)	1 (<1%)	3 (1%)
Malaria rapid test, positive out of total tested		9/125 (7%)	12/91 (13%)	21/216 (10%)
Contact with domestic animals		16 (6%)	18 (9%)	34 (7%)
Contact with wild animals		2 (1%)	4 (2%)	6 (1%)
**Employment**				
Farmer		72 (25%)	11 (5%)	83 (17%)
Student		40 (14%)	39 (19%)	79 (16%)
Children below the local school age*		91 (31%)	112 (55%)	203 (41%)
Craftsman or merchant		21 (7%)	6 (3%)	27 (5%)
Military		1 (<1%)	10 (5%)	11 (2%)
Public server		1 (<1%)	3 (1%)	4 (1%)
Unemployed		37 (13%)	5 (2%)	42 (9%)

* The typical starting age for school in the study area is 6 years old.

**Figure 1:**
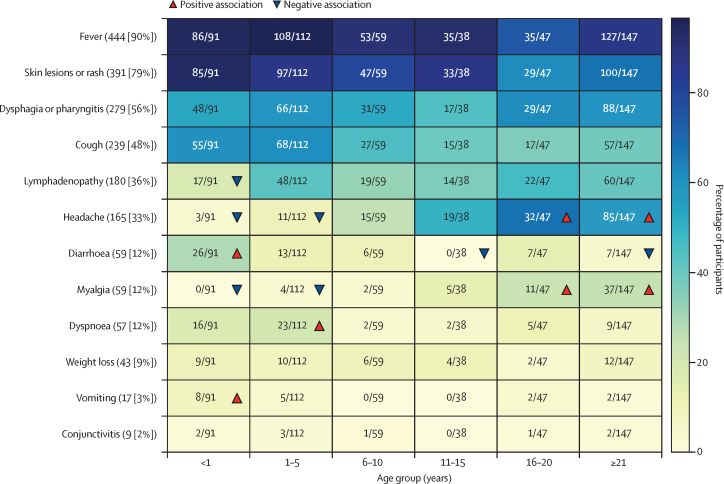
Prevalence of symptoms and signs of the PCR positive MPXV cohort Counts in descending order of 12 mpox-related symptoms or signs across the 494 participants and all age groups. The heat map shows the number of participants in different age groups (x-axis) who were found to present symptoms and signs of mpox (y-axis). The cells are coloured by the proportion of participants belonging to each age group that has the symptom. Red upward triangles indicate a significant positive association between the symptom and the age group while blue downward triangles indicate a significant negative association, based on standardised chi-square residuals (residual ≥1·96). Headache and myalgia were recorded with lower frequency in participants aged younger than 1 and 1–5 years. The absence of these symptoms likely reflects limitations in symptom assessment within these age groups rather than a true absence of clinical manifestation. Fever was defined as ≥38°C. MPXV=monkeypox virus.

The most prominent symptom or sign observed was fever (444 [90%] of 494 participants), followed by skin rash or lesions (391 [79%]), dysphagia or pharyngitis (279 [56%]); figure 1). Conjunctivitis was observed in nine (2%) participants. Participants less often presented with symptoms or signs of diarrhoea, myalgia, dyspnoea, weight loss, conjunctivitis, and vomiting. Among participants younger than 1 year, diarrhoea and vomiting occurred more frequently than in other age groups, whereas lymphadenopathy, headache, and myalgia were less common than in other age groups. Among participants aged 1–5 years, dyspnoea was more frequent, and headache and myalgia were less frequent. In participants aged 11–15 years, diarrhoea was uncommon. Among those aged 16–20 years, headache and myalgia occurred more often. In participants aged 21 years or older, diarrhoea was less frequent, whereas headache and myalgia were more common. No statistical differences were found in the symptom count average among different age groups (appendix 4 p 9). We included the counts of each symptom in male and female participants of different age groups in appendix 4 (p 10).

**Figure 2:**
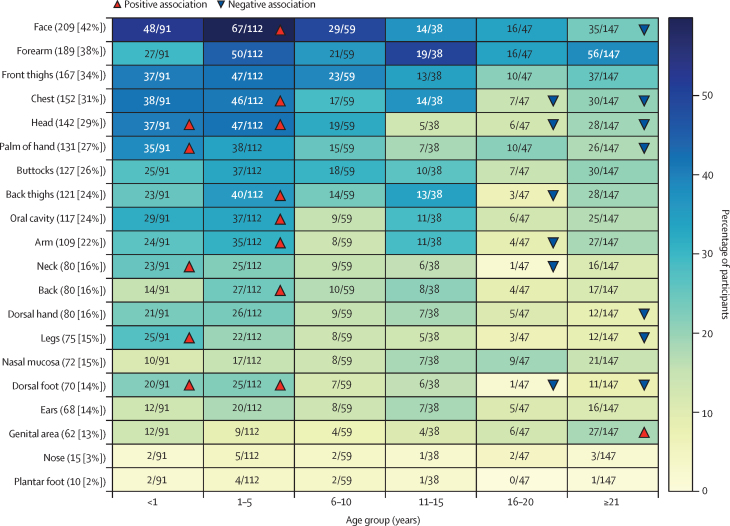
Presence of lesions in different anatomical regions Counts in descending order of lesions in 20 different anatomical regions across all age groups. The heat map shows the number of participants in different age groups (x-axis) who had lesions in different anatomical regions (y-axis). The cells are coloured by the proportion of participants belonging to each age group. Red upward triangles indicate a significant positive association between the presence of lesions and the age group while blue downward triangles indicate a significant negative association, based on standardised chi-square residuals (residual ≥1·96).

Enrolled participants presented with a full range of skin lesions, from rash to healing crusts, which were observed in various anatomical regions. 395 (80%) of the 494 participants who were PCR positive presented at the hospital with lesions in at least one anatomical region. The highest proportion of individuals presented with lesions on the face (209 [42%]), forearm (189 [38%]), anterior aspect of thighs (167 [34%]), and chest (152 [31%]; figure 2). In participants aged younger than 1 year, significantly higher frequencies of lesions were observed on the head (37 [41%] of 91), neck (23 [25%]), palm of hand (35 [38%]), legs (25 [27%]), and dorsal foot (20 [22%]). We also found a higher frequency of lesions on the head (47 [42%] of 112), face (67 [60%]), back (27 [24%]), arm (35 [31%]), chest (46 [41%]), posterior aspect of thighs (40 [36%]), dorsal foot (25 [22%]), and oral cavity (37 [33%]) in participants aged 1–5 years. By contrast, we found lower frequencies of lesions on the head (six [13%] of 47), neck (one [2%]), arm (four [9%]), chest (seven [15%]), posterior aspect of thighs (three [6%]), and dorsal foot (one [2%]) in participants aged 16–20 years. In participants aged 21 years or older, lower frequencies were also found in the head (28 [19%] of 147), face (35 [24%]), dorsal hand (12 [8%]), palm of hand (26 [18%]), chest (30 [20%]), legs (12 [8%]), and dorsal foot (11 [7%]), and a higher frequency of lesions in the genital area (27 [18%]).

**Figure 3:**
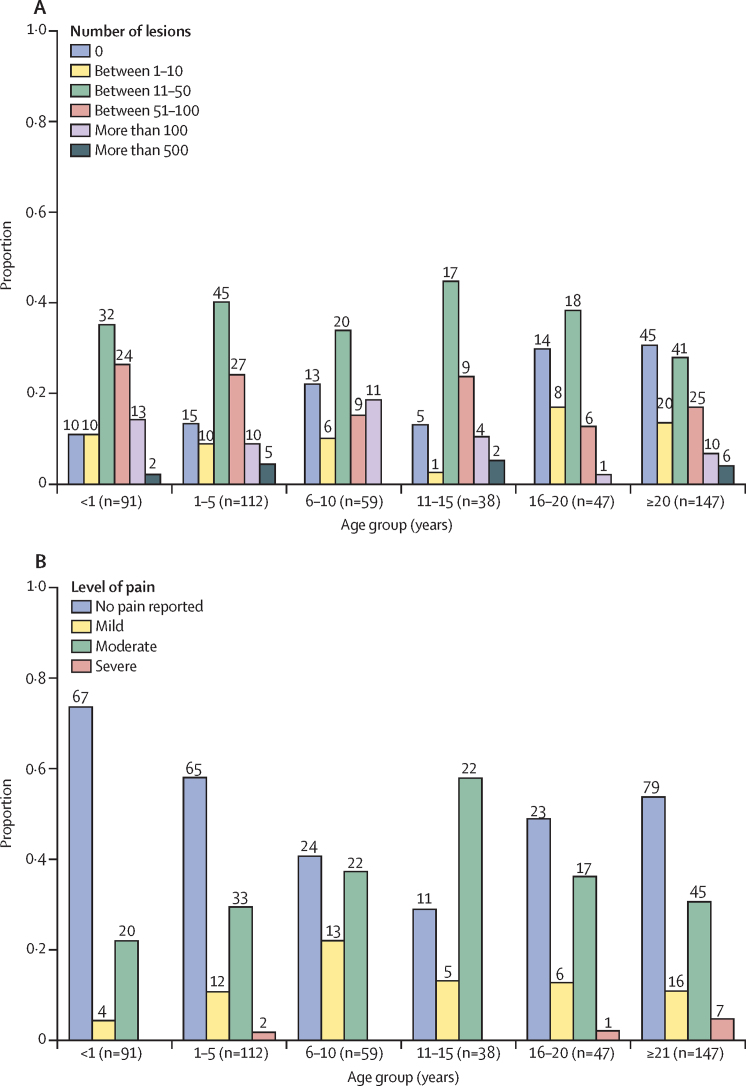
Total number of lesions (A) and level of pain (B) (A) Proportion of participants of different age groups with a pre-established range of total body lesion count, represented by each of the bars. Above each bar, we included the numerator and the denominator used for calculating the proportions plotted. (B) Proportions (y-axis) of participants of different age groups (x-axis) according to the level of pain per hospital staff assessment, represented by each of the bars. Above each bar, we included the numerator and the denominator used for calculating the proportions plotted.

We found most participants had several regions throughout the body presenting with clusters of lesions where the mean number of anatomical regions with mpox lesions per individual was 4·2 (SD 3·7; median 4 [IQR 2–6]). The mean number of anatomical regions with lesions was generally significantly higher in children aged between 0 years and 5 years than in individuals older than 15 years (appendix 4 p 11). We include the proportion of each anatomical region with lesions, stratified by age and sex, in appendix 4 (p 12). The total number of body lesions was determined per individual and analysed for each of the different age groups (figure 3A). The most common total number of body lesions was between 11 and 50 across all age groups. Hospital staff assessed the level of pain (mild, moderate, and severe) associated with the presence of lesions. In all age groups, moderate level of pain was predominant, with the participants feeling tolerable pain with the palpation of lesions. Ten participants (two aged 1–5 years; one aged 16–20 years; and seven aged ≥21 years) were assessed with severe pain where the participants showed intense response to even minimal palpation. Touching the lesion triggered immediate and strong reactions such as withdrawal, aggression, vocalisation, or escape attempts (figure 3B).

**Figure 4:**
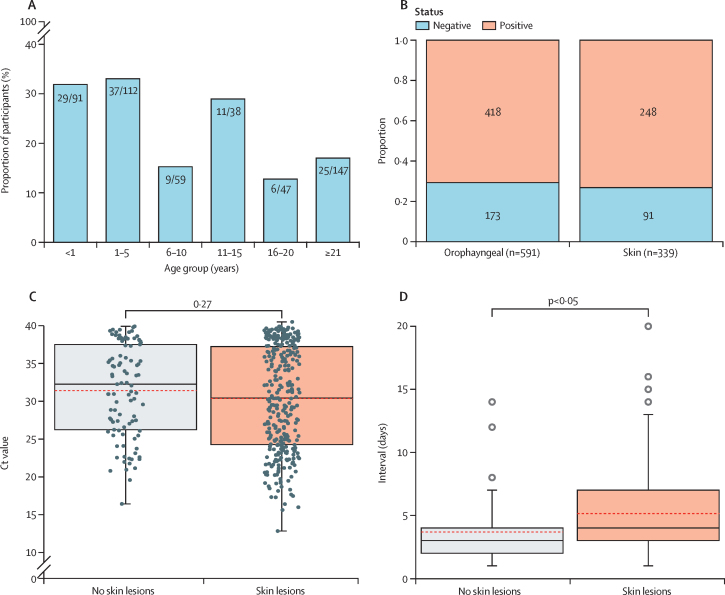
Comparison of mpox lesions in the skin and oral cavity (A) The percentage of participants in different age groups who were observed with lesions in the oral cavity or oropharynx (including the regions mouth, sublingual, oropharynx, and pharynx). Each bar represents an age group, and it is annotated with the numerator and denominator used to count for the percentages. (B) We counted the proportion of qPCR reactions extracted from the skin and oral cavity. A Ct value of 40 or less was considered positive. Each stacked bar represents the positivity and negativity rate of skin and oral cavity. Inside each bar, we include the numerator and denominator that indicates the presence of lesions in each region. (C) We compared the oral cavity Ct values of participants with and without skin lesions. The Ct value variable was found to be non-parametric in both groups (Shapiro–Wilk p values <0·0001 and 0·0001 for the groups with and without skin lesions, respectively) and no statistical differences were found in the medians of the two groups (Mann–Whitney *U* test p value=0·27). (D) We compared the difference in time from symptom onset to hospital presentation in participants with skin lesions and a positive skin swab versus participants with no skin lesion and a positive oral cavity swab. The time variable was found to be non-parametric in both groups (Shapiro–Wilk p values <0·0001) and its median was significantly lower in participants with no skin lesions (Mann–Whitney *U* test p value <0·0001). Ct values distribution and symptom profile of age group-specific participants are included in appendix 2 (pp 15–17). We also describe the age-group Ct value of skin swabs of participants with skin lesions or a rash (appendix 2 p 18). Ct=Cycle threshold.

117 participants (24%) had lesions in the oral cavity or oropharynx. The highest incidence of oral cavity lesions was found in those younger than 1 year (29 [32%] of 91), aged 1–5 years (37 [33%] of 112), and aged 11–15 years (11 [29%] of 38; figure 4A). The overall PCR positivity rate was similar in swabs of the oral cavity or oropharynx (418 [71%] of 591) and in skin (248 [73%] of 339; figure 4B). We subset our data into oral swabs as follows: (1) participants with skin lesions (median cycle threshold [Ct] 30·14 [IQR 24·27–37·23]) and (2) oral swabs of participants with no skin lesions (32·26 [26·24–37·23]). The median Ct values did not yield significant differences (figure 4C). Further distribution and modality of Ct values in age groups and the symptom profile can be found in appendix 4 (pp 16–17), where participants with no skin lesions showed a higher frequency of dysphagia and pharyngitis. Finally, the time from symptom onset to hospital presentation was significantly lower in participants with no skin lesions (3 days [2–4]) than in participants with skin lesions (4 days [3–7]; figure 4D). The higher viral load in swabs from skin lesions might be explained by these participants having had enough time for skin lesions to arise, potentially indicating a more advanced course of the infection. We also found that 11 participants who initially had no skin lesions later developed skin lesions or a rash (mean 10·41 days [SD 5]).

There were four fatalities during the study period, which totals a case-fatality rate of 0·8% in the PCR positive cohort and 1·3% in children and adolescents (≤15 years). All four fatalities had concomitant severe malaria. A 4-month-old girl had anaemia and severe malaria. The participant's condition worsened rapidly, leading to respiratory failure and ultimately death. A 6-month-old boy presented with widespread pustular skin eruptions (evolving for 1 week), anaemia, and severe malaria. On examination, the participant was in poor general condition with respiratory distress (rapid breathing), pale conjunctiva, pale palms and soles, and a cough. The clinical course was marked by progressive deterioration, followed by oxygen desaturation and ultimately death. A 17-month-old boy presented with fever and skin eruptions over 5 days and severe malaria with hyperparasetaemia. Examination revealed widespread pustules, vesicles, and papules across the body. The condition progressed to renal and liver complications ultimately resulting in death. A 6-year-old girl presented with fever, skin eruptions (evolving over 4 days), anaemia, and severe malaria. The participant was initially treated at a different health centre without improvement. Clinical findings on presentation at Lwiro Hospital included pustular skin lesions, rhonchi in both lung fields, oxygen desaturation, and pitting oedema in the lower limbs. A few hours after admission, the participant had a cardiorespiratory arrest, leading to death. No fatalities were reported in the 13 pregnant participants.

**Figure 5:**
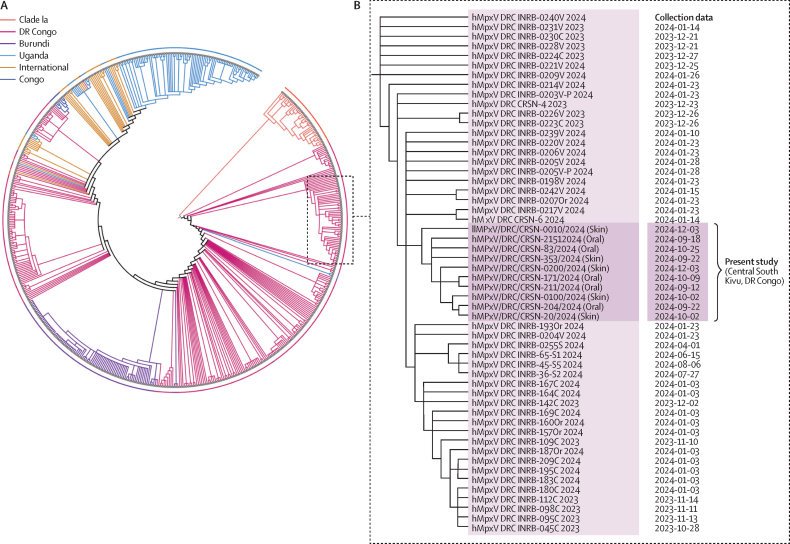
Phylogenetic tree of the ten subclade Ib/2023sh MPXV sequences obtained in this study (A) A circular representation of a phylogenetic tree consisting of both subclade Ia and Ib sequences. Subclade sequences are reported from Burundi, DR Congo, Congo (Brazzaville), Uganda, and international (travel-associated). (B) The expanded rectangular view of the phylogenetic tree that consists of the ten sequences reported in this study. Sequences reported in the study are shown with a dark background, while early subclade Ib sequences from South Kivu are indicated with a light background. Collection dates are based on the metadata available on the Global Initiative on Sharing All Influenza Data repository and include the anatomical region where the swabs were obtained (skin or oral cavity).

Nanopore long-read sequencing was successfully applied to genotype MPXV subclade Ib/2023sh from five skin lesion swabs and five oropharyngeal swabs from ten study participants younger than 5 years. Details of the sequencing runs are included in appendix 4 (pp 13–14). MPXV subclade Ib/2023sh consensus genomes were obtained for all samples. A combined phylogenetic tree of clade I sequences (Ia and Ib) showed that the ten genomes reported in the present study from central South Kivu make a cluster with the early subclade Ib/2023sh sequences reported from eastern DR Congo between 2023 to early 2024 (figure 5). These results suggested that the MPXV circulating in the central South Kivu outbreak descends from the early MPXV subclade Ib/2023sh viruses found in Kamituga, South Kivu, eastern DR Congo, where the first documented subclade Ib sequence was isolated.

## Discussion

The typical paediatric participant in our cohort presented to the hospital with fever and skin rash and lesions. Participants aged 0–5 years often had a higher frequency of lesions on the head, face, neck, back, arm, palm of hand, chest, anterior aspect of thighs, legs, dorsal foot, and oral cavity. The most common symptoms and signs identified in this cohort are in line with previous clinical descriptions of clade I that include rash and skin lesions listed as essentially universal in confirmed cases of mpox clade I.^12^, ^15^, ^17^ Fever, lymphadenopathy, myalgia, headache, and sore throat or pharyngitis are also commonly reported symptoms. We also identified a higher proportion of lesions in the genitals of adult individuals. In previously described mpox subclade Ib/2023sh cohorts,^12^, ^15^, ^17^, ^18^ genital lesions were frequently observed, consistent with transmission associated with sexual contact. The affected populations largely consisted of sexually active adults, including sex workers, who were part of the initial transmission networks of the 2023 outbreak. Here, we found that children and adolescents (≤15 years) comprised 61% (300 of 494) of the PCR-positive participants. Given that sexual transmission is highly unlikely in this predominantly paediatric population, the observed epidemiological patterns suggest that non-sexual routes of exposure could have contributed to the transmission of MPXV subclade Ib/2023sh in South Kivu. However, the exact transmission pathways have not been precisely determined from our data and will be explored in future investigations.

The households in this study were crowded (median six people, including a median of four individuals aged ≤15 years), suggesting living conditions in which close physical contact among children and adolescents is frequent. Children are recognised as important drivers of viral circulation due to their high frequency of close physical contact, limited awareness of personal hygiene, and less well defined physical boundaries in social interactions.^20^, ^21^, ^22^, ^23^ The routine sharing of beds, clothing, towels, and sanitation facilities might increase opportunities for non-sexual contact and potential environmental contamination. These findings are consistent with reports from other cohorts,^17^ where children and adolescents often present with extragenital lesions and household transmission was suspected. Although the relative contribution of direct contact, caregiving practices, and fomite exposure remains uncertain, the household factors we identified provide a plausible explanation for the clustering of paediatric cases observed. Further investigation is needed to better delineate transmission pathways in a household setting, particularly given that sexual and non-sexual routes might exist within the same household.

The clinical presentation of mpox in children and adolescents in our cohort aligns with descriptions from other MPXV subclade Ib/2023sh reports, particularly with respect to the predominance of disseminated extragenital lesions in paediatric cases, overall symptomatology, and similar case-fatality rates. The cohort described by Brosius and colleagues^17^ represents an important clinical and epidemiological comparative framework for MPXV subclade Ib/2023sh circulation in South Kivu. Their cohort was primarily adult, with only 25 of 510 cases occurring in children younger than 5 years, and sexual exposure was commonly identified among affected adults whereas paediatric infections were attributed largely to household transmission. By contrast, our cohort is skewed towards children and adolescents, and clear exposure pathways were not ascertained. Despite these differing age structures, both cohorts reported a similar pattern in lesion distribution with genital involvement concentrated among adults with presumed sexual exposure and more generalised lesions among children and adolescents, suggesting a consistent phenotype across outbreaks. Overall, the clinical phenotype and lesion distribution seems to be directly associated with the route of exposure. The distinct age distribution in our cohort could be shaped by underlying socioeconomic and behavioural differences between study sites. Previous MPXV subclade Ib/2023sh cohorts were based in Kamituga, a mining town with established commercial sex networks and higher likelihood of adult sexual exposure. By contrast, Miti-Murhesa is an agrarian community with fewer commercial sex establishments, greater maternal involvement in field labour, and household structures that place children and adolescents in closer, prolonged contact with caregivers and siblings. These contextual factors might contribute to a different pattern of human-to-human transmission and help explain the predominance of paediatric cases observed in our setting.

Our cohort of participants is based on individuals who presented to the hospital, which is a limitation as this design might not have captured the full epidemiological picture of mpox infections in the community. There were reports that fear of stigma and privacy concerns were found to deter people from presenting for care in the 2022 mpox multicountry outbreak.^24^ In this study, we only obtained samples and data from patients during admission. Additional longitudinal studies in hospitalised patients and the community are required to continue exploring the clinical progression of mpox over time and explore the rate of positivity of skin and oral cavity swabs. Our study also was not designed to capture the mode of transmission both in hospitalised patients and in the community; therefore, it does not allow us to better understand the modes of MPXV subclade Ib/2023sh transmission. The viral load of oral swabs of participants with and without skin lesion was similar. Our inclusion criteria allowed us to recruit participants without skin lesions if they had been in contact with an individual with suspected mpox in the previous 21 days and had fever, dysphagia or pharyngitis, or cervical lymphadenopathy. The lower incidence of skin lesions in our recruited participants is likely linked to the chronological course of mpox infection. Other reports identified a median of approximately 7 days for participants to develop skin lesions,^9^, ^17^ whereas our participants with no skin lesions presented to the hospital within 3 days of symptom onset. Still, we cannot rule out the involvement of technical issues such as low template concentration or contamination, especially in a resource-limited setting amid a public health emergency. We acknowledge that the existence of additional factors such as the high rate of malnutrition^25^ and ongoing conflict in eastern DR Congo^26^ might create suitable scenarios for co-infections,^27^ exacerbating the morbidity of mpox. In fact, the logistical constraints brought by the conflict in South Kivu did not allow us to molecularly test all recruited participants. To provide the full demographics and epidemiological details of all participants, and not only those who tested positive, we included the data of all recruited participants in appendix 4 (pp 2–3). Despite varicella not circulating in the study area during the study period, the lack of molecular diagnostics does not allow us to rule out that varicella zoster virus was a cofounding variable. In conclusion, we identified children and adolescents as important targets of MPXV subclade Ib/2023sh infection who might therefore drive transmission given crowded household conditions and well known disregard by children for personal space. Since most households in our study area were made up of families with two-thirds of their occupants being children and adolescents (aged ≤15 years) and given the high birth rate in central Africa (especially the Kivu region), we might expect a constant influx of mpox-naive individuals in the absence of robust yearly vaccination efforts, potentially resulting in recurrent MPXV outbreaks.

### Contributors

### Data sharing

The ten MPXV sequences obtained in this study are deposited in Global Initiative for Sharing All Influenza Data (GISAID); their accession numbers are listed in appendix 4 (pp 13–14). The findings of this study are based on metadata associated with 387 MPXV subclade Ib/2023sh sequences available on GISAID up to May 24, 2025, via EPI_SET_250524qc, and accessible at https://doi.org/10.55876/gis8.250524qc. De-identified individual participant clinical, epidemiological, and demographic data will be made available upon reasonable request for the purpose of academic, non-commercial analyses. A data dictionary defining each field in the dataset will also be made available as well as the study protocol. Upon approval, data access will be provided without investigator support through a secure transfer. Data will be available from the date of publication for up to 5 years. Requests for access should be directed to the corresponding author.

## Declaration of interests

GSM, AK, MD, and DJK are shareholders of the company BioForge Canada Limited. BioForge Canada Limited is a company that uses bioinformatics in immunological approaches in the monitoring, prevention, and treatment of infectious diseases. The authors disclose that the interests of BioForge Canada Limited had no impact in this study. DJK is a scientific advisory board member for Emergent BioSolutions. BA-R received honoraria for participation in live meetings from Sanofi Pasteur France and Sanofi Pasteur Canada related to pertussis and respiratory syncytial virus, received nominal payment as a reviewer for Elsevier and as a member of a data and safety monitoring board for a study conducted by Chulalongkorn University (Bangkok, Thailand); and is co-investigator on studies funded by GSK, Pfizer, Merck, Moderna, Vaccitech, and Inventprise. All funds have been paid to his institute, and he has not received any personal payments. All other authors declare no competing interests.

## Supplementary Materials

French translation of the abstract

Swahili translation of the abstract

Mashi translation of the abstract
