# Supplementary material for: Case presentation of patients hospitalised with mpox (subclade Ib/2023sh) including children, adolescents, and adults in South Kivu, Democratic Republic of the Congo: an observational cohort study
Source: Lancet Infect Dis. 2026 Jun;26(6):590–600. doi: 10.1016/S1473-3099(26)00051-4 (PMC13241576; doi:10.1016/S1473-3099(26)00051-4)
Supplement: French translation of the abstract [file mmc1.pdf]

THE LANCET
Infectious Diseases

Supplementary appendix 1

This translation in French was submitted by the authors and we reproduce it as supplied. It has not been peer reviewed. *The Lancet*'s editorial processes have only been applied to the original in English, which should serve as reference for this manuscript.

Cette traduction en français a été proposée par les auteurs et nous l'avons reproduite telle quelle. Elle n'a pas été examinée par des pairs. Les processus éditoriaux du *Lancet* n'ont été appliqués qu'à l'original en anglais et c'est cette version qui doit servir de référence pour ce manuscrit.

Supplement to: Flores Girón L, Sganzerla Martinez G , Daniel BN, et al. Case presentation of patients hospitalised with mpox (subclade Ib/2023sh) including children, adolescents, and adults in South Kivu, Democratic Republic of the Congo: an observational cohort study. *Lancet Infect Dis* 2026; published online March 9. https://doi.org/10.1016/S1473-3099(26)00051-4.

Résumé

Contexte

La mpox constitue un problème de santé publique dans l'est de la République démocratique du Congo (RDC). Elle continue de provoquer un nombre important d'hospitalisations, avec une évolution du profil démographique incluant des enfants et des adolescents, ce qui nécessite une investigation clinique et épidémiologique approfondie. Dans cette étude, nous visons à décrire les caractéristiques cliniques des patients hospitalisés atteints d'une infection par le virus de la variole du singe (MPXV), sous-clade Ib/2023sh, dans le territoire de Kabare, au Sud-Kivu, en RDC.

Méthodes

Cette étude de cohorte observationnelle a inclus des patients admis avec une suspicion de mpox au centre de référence pour le traitement de la mpox à l'Hôpital de Lwiro, au Sud-Kivu (RDC). Les participants éligibles devaient présenter, au moment de l'inclusion, des lésions cutanées compatibles avec l'infection. Les individus ne présentant pas de lésions compatibles avec une infection par le MPXV étaient également éligibles s'ils présentaient au moins l'un des symptômes suivants : fièvre, adénopathie cervicale ou pharyngite, à condition qu'ils aient été en contact avec une personne suspectée de mpox au cours des 21 derniers jours. Les données issues des dossiers hospitaliers et de formulaires cliniques standardisés ont permis de recueillir des informations démographiques, les symptômes et signes cliniques à l'admission, les issues cliniques, ainsi que les caractéristiques générales des patients. Des analyses descriptives ont été utilisées pour résumer les profils cliniques et épidémiologiques des participants présentant une confirmation moléculaire du MPXV sous-clade Ib/2023sh.

Résultats

Entre le 3 août 2024 et le 8 février 2025, le MPXV sous-clade Ib/2023sh a été détecté chez 494 (77 %) des 643 participants, avec un âge médian de 9 ans (IQR 2–24). Les participants positifs pour le MPXV sous-clade Ib/2023sh étaient plus souvent de sexe féminin (290 [59 %]) et généralement plus âgés (l´âge médian était de 16 ans [4–25]) que les participants de sexe masculin (204 [41 %] ; l´âge médian était de 4 ans [1–14]). Parmi les 494 participants, 300 (61 %) étaient âgés de 15 ans ou moins. La fièvre (444 [90 %]), les lésions cutanées ou éruption (391 [79 %]) et la dysphagie (279 [56 %]) étaient les symptômes les plus fréquents. Les enfants âgés de 0 à 5 ans présentaient plus fréquemment des lésions au niveau de la tête (84 [41 %] sur 203), du visage (67 [33 %]), du cou (23 [11 %]), du dos (27 [13 %]), du bras (35 [17 %]), de la paume de la main (35 [17 %]), du thorax (46 [23 %]), de la face postérieure des cuisses (40 [20 %]), des jambes (25 [12 %]), du dos du pied (45 [22 %]) et de la cavité buccale (37 [18 %]). Au total, 117 (24 %) des participants présentaient des lésions dans la cavité buccale. Les écouvillons de la cavité buccale et de l'oropharynx ont permis de détecter le MPXV sous-clade Ib/2023sh même en l'absence de lésions cutanées exploitables.

Interprétation

La proportion élevée d'enfants et d'adolescents (≤15 ans) distingue notre cohorte des autres descriptions cliniques du nouveau sous-clade Ib/2023sh du MPXV. Nous émettons donc l'hypothèse d'un changement démographique dans la population touchée contribuant à la propagation communautaire de la mpox dans la région du Sud-Kivu, en RDC. Les mesures de santé publique devraient tenir compte de la nécessité de réduire la transmission chez les enfants et les adolescents.
