# Supplementary material for: Case presentation of patients hospitalised with mpox (subclade Ib/2023sh) including children, adolescents, and adults in South Kivu, Democratic Republic of the Congo: an observational cohort study
Source: Lancet Infect Dis. 2026 Jun;26(6):590–600. doi: 10.1016/S1473-3099(26)00051-4 (PMC13241576; doi:10.1016/S1473-3099(26)00051-4)
Supplement: Swahili translation of the abstract [file mmc2.pdf]

THE LANCET
Infectious Diseases

Supplementary appendix 2

This translation in Swahili was submitted by the authors and we reproduce it as supplied. It has not been peer reviewed. *The Lancet*'s editorial processes have only been applied to the original in English, which should serve as reference for this manuscript.

Tafsiri hii katika Swahili iliwasilishwa na waandishi na tunatengeneza tena kama hutolewa. Haijapitiwa. Mchakato wa hariri wa Lancet umetumika tu kwa asili kwa Kiingereza, ambayo inapaswa kutumika kama kumbukumbu kwa muswada hii.

Supplement to: Flores Girón L, Sganzerla Martinez G , Daniel BN, et al. Case presentation of patients hospitalised with mpox (subclade Ib/2023sh) including children, adolescents, and adults in South Kivu, Democratic Republic of the Congo: an observational cohort study. *Lancet Infect Dis* 2026; published online March 9. https://doi.org/10.1016/S1473-3099(26)00051-4.

Muhtasari

Asili

Ugonjwa wa **mpox** ni tatizo kubwa la afya ya jamii katika mashariki ya Jamhuri ya Kidemokrasia ya Kongo (RDC). Ugonjwa huu unaendelea kusababisha wagonjwa wengi kulazwa hospitalini, na sasa tunaona pia mabadiliko katika kundi la watu wanaoathirika, likiwa linajumuisha watoto na vijana. Hali hii inaonyesha umuhimu wa kufanya uchunguzi wa kina wa kitabibu na wa magonjwa. Katika utafiti huu tunalenga kuelezea hali ya kitabibu ya wagonjwa waliolazwa hospitalini walioambukizwa virusi vya monkeypox (MPXV), sous-clade Ib/2023sh, katika eneo la Kabare, Sud-Kivu, RDC.

Mbinu

Utafiti huu wa kikundi cha wagonjwa (cohorte observationnelle) ulihusisha wagonjwa waliolazwa hospitalini wakiwa na tuhuma ya mpox katika kituo cha rufaa cha matibabu ya mpox katika Hospitali ya Lwiro, Sud-Kivu (RDC). Washiriki waliokubaliwa katika utafiti walipaswa kuwa na vidonda vya ngozi vinavyofanana na mpox wakati wa kuingia katika utafiti. Watu ambao hawakuwa na vidonda hivyo waliweza pia kujumuishwa ikiwa walikuwa na angalau dalili moja kati ya hizi: homa, kuvimba kwa tezi za shingo, au maumivu ya koo (pharyngite), na kama walikuwa wamekutana na mtu anayeshukiwa kuwa na mpox ndani ya siku 21 zilizopita.
Taarifa zilikusanywa kutoka katika mafaili ya hospitali na fomu za kitabibu zilizotayarishwa kwa ajili ya utafiti. Taarifa hizi zilihusisha sifa za kidemografia, dalili na ishara za wagonjwa wakati wa kuingia hospitalini, matokeo ya ugonjwa, pamoja na hali ya jumla ya wagonjwa. Uchambuzi wa takwimu za maelezo ulitumika kuelezea sifa za kitabibu na za epidemiolojia za washiriki waliothibitishwa kwa vipimo vya maabara kuwa na MPXV sous-clade Ib/2023sh.

Matokeo

Kati ya tarehe 3 Agosti 2024 na 8 Februari 2025, MPXV sous-clade Ib/2023sh iligunduliwa kwa washiriki 494 (77%) kati ya washiriki 643, wakiwa na umri wa kati wa miaka 9 (IQR 2–24). Washiriki waliokuwa na MPXV sous-clade Ib/2023sh walikuwa mara nyingi wanawake (290 [59%]) na kwa kawaida walikuwa wakubwa zaidi kwa umri (umri wa kati miaka 16 [4–25]) kuliko wanaume (204 [41%]; umri wa kati miaka 4 [1–14]).
Kati ya washiriki 494, jumla ya 300 (61%) walikuwa na umri wa miaka 15 au chini. Homa (444 [90%]), vidonda au upele wa ngozi (391 [79%]) na ugumu wa kumeza chakula (dysphagia) (279 [56%]) zilikuwa dalili zilizoonekana mara nyingi. Watoto wa miaka 0 hadi 5 walionyesha vidonda mara nyingi zaidi katika sehemu mbalimbali za mwili kama vile kichwa, uso, shingo, mgongo, mikono, viganja, kifua, nyuma ya mapaja, miguu, juu ya mguu na ndani ya mdomo. Kwa jumla, washiriki 117 (24%) walikuwa na vidonda ndani ya mdomo. Sampuli zilizochukuliwa kwa usufi ndani ya mdomo na katika oropharynx ziliweza kugundua MPXV sous-clade Ib/2023sh hata wakati hakukuwa na vidonda vya ngozi vinavyoweza kuchunguzwa.

Ufafanuzi

Idadi kubwa ya watoto na vijana (≤15 miaka) inaonyesha tofauti kati ya kikundi chetu cha utafiti na maelezo mengine ya kitabibu kuhusu sous-clade mpya Ib/2023sh ya MPXV. Kwa hiyo tunadhani kwamba kuna mabadiliko ya kidemografia katika kundi la watu wanaoathirika ambayo yanachangia kuenea kwa mpox katika jamii katika eneo la Sud-Kivu, RDC. Hatua za afya ya umma zinapaswa kuzingatia umuhimu wa kupunguza maambukizi miongoni mwa watoto na vijana.
