# Supplementary material for: Case presentation of patients hospitalised with mpox (subclade Ib/2023sh) including children, adolescents, and adults in South Kivu, Democratic Republic of the Congo: an observational cohort study
Source: Lancet Infect Dis. 2026 Jun;26(6):590–600. doi: 10.1016/S1473-3099(26)00051-4 (PMC13241576; doi:10.1016/S1473-3099(26)00051-4)
Supplement: Mashi translation of the abstract [file mmc3.pdf]

THE LANCET
Infectious Diseases

Supplementary appendix 3

This translation in Mashi was submitted by the authors and we reproduce it as supplied. It has not been peer reviewed. *The Lancet*'s editorial processes have only been applied to the original in English, which should serve as reference for this manuscript.

Ekihindurwa kino mu Mashi cyatanzwe n'abanditsi b'inyandiko kandi turagisubiza hano nk'uko cyatanzwe. Nticyasuzumwe n'abasuzuma b'inzobere mu mwuga (peer review). Uburyo bw'inyandiko bw'umwimerere mu Cyongereza bwo bwonyine nibwo bwakurikijwe n'ubuyobozi bwa The Lancet, kandi bwo bugomba gufatwa nk'ishingiro ry'iyi nyandiko.

Supplement to: Flores Girón L, Sganzerla Martinez G , Daniel BN, et al. Case presentation of patients hospitalised with mpox (subclade Ib/2023sh) including children, adolescents, and adults in South Kivu, Democratic Republic of the Congo: an observational cohort study. *Lancet Infect Dis* 2026; published online March 9. https://doi.org/10.1016/S1473-3099(26)00051-4.

ENSHULA NYANZI

Endwala ya mpox kali kadali kanene omukalamo k'abantu b'omu KONGO. Obulwala bugendekire bwaluza bantu mwandu omu bitaro. Na ntyo rhulirhwabona empindulo oku bantu baherhe obo bulwala, lero bubonekine bwenene n'oku bana n'oku misole. N'okûla kuyerekine oku kulikwinja oku longereza eyi ndwala (epidemiologia) lyo bamanya bwinja gurhi eyi ndwala egendekire ya landa. N'oku kulongereza, rhwalonza rhumanyise oborhere bw'abalwala bayambukiragwa ne virus ya monkey pox(MPXV), ci bwenene ehemagalwa sous-clade Ib/2023sh omu teritoire y'e kabare, sud-kivu, RDC.

OKU RHAKAGIKOLA

Obu bulongerizi bwali lolire abalwala bali omubitaro abali bikeebwe oku baherhe mpox omubitaro bye Lwiro aha babukira mpox omu Sud-kivu RDC. Abantu bali yemerirwe okuja m'obo bulongerizi, bali balya baherhe ebihulu okuluhu lw'omubiri gwabo bishushine n'endwala ya Mpox amango g'okuja omubitaro. N'abandi barhahêrhe ebyo bihulu baliyankirwe omu bulongerizi erhi bakaba baherhe ebimanyiso bya ntya: ishushira ,ukurhunda, emburho y'omubiri g'omwigosi, okulumwa k'omumiro. Ci kone kwalihunirwe oku manya erhi yâli shimanine, erhi kuhumana ko n'omuntu olwazire mpox omu kagarhi k'ensiku 21 zagerega. Emyanzi y'abalwala yakagi rhengerera omu: Makaratasi y'omulwala, N'omu formulaire y'obushimbulizi b'obulwala. Emyanzi cigushe yalintya: emyaka n'okumanya erhi mulume erhi munyere, ebimanyiso by'endwala amango bajaga omu bitaro, Ishuzo ly'amafumu, N'oborhere bwabalwala boshi. Enyuma yahoo, rhwalonza okumanya bwinja gurhi omulwala ajayosire kuli ngasi yeshi wabukagwa

EBYALINGAGWA

Kurhenga omu n'siku 03 mwezigwa munani 2024 kuhika onu n'siku 08 Mwezigwa kabiri 2025, Virus Mpxv Sous Clade 1b/2023 sh bwabonekine omubantu 494 (77%) okubantu 643 abali omu bulongerizi. Emyaka y'amango g'obulwala yali y'omumyaka mwenda. Abantu babonekaga mweyindwala (Virus) bali bakazi 290 (59%) na balume bali 204 (41%). Abakazi baherhe endwala bali omu myaka ya aha kagarhi ka 16 nabo abalume bali omu kagarhi ka myaka 4. Omu balwala 494, boshi 300 (61%) bali bana b'emisole ba myaka 15 erhi idako. Ebimanyiso byabonekanaga bwenene neyindwala byali byebi : Ishunshira, balwala 444 (90%); ebihulu erhi amahere g'okuluhu lwomubiri balwala 391 (79%); okurhagalilya ebiryo, balwala 279 (56%). Okubana ba myaka 0 Kuhika myaka 5, ebihulu byakagi bonekana byenene kwezi nafasi z'omubiri: irhwe (84 [41%] omu 203), amalanga (67[33%]), Igosi 23 [11%]), omugongo (27 [13%]), amaboko (35 [17%]), ebigasha bya amaboko (35 [17%]), echifuba (46 [23%]); enyuma y'eciberu (40 [20%]); amagulu (25 [12%]); emugongo g'olushando lwamagulu (45 [41%]), omukanwa (37 [18%]). Omu byoshi, 117 (24%) bali n'ebihulu by'ekanwa. Omurhi guli kolwafu omukanwa n'omumumiro zalibonekine MPXV Sous Clad 1b/2023 sh, chiro n'amango harhaliherhe bihulu byokuluhu l'omubiri.

OKUHUGULIRA

Okwigana linji lyabana n'emisole (≤15 ans). Omucikundi chirhu chabanganga n'eyindi myanzi ye citabu cilolire sous clad mpyahya 1b / 2023 sh ya %MPXV. Rhuhabwire obulyo bwa empindulo y'edemografia omu bantu b'okuyahukizana. Oko kwarhumire abantu bayahukirwa na Mpox omu mulala n'omu chihugo cha sud-Kivu RDC. Abafumu boba munganga abo, bahunirwe bachihangane okulwisa okuyahukiza eyo ndwala omu bana n'omu misole.
