# Supplementary material for: Case presentation of patients hospitalised with mpox (subclade Ib/2023sh) including children, adolescents, and adults in South Kivu, Democratic Republic of the Congo: an observational cohort study
Source: Lancet Infect Dis. 2026 Jun;26(6):590–600. doi: 10.1016/S1473-3099(26)00051-4 (PMC13241576; doi:10.1016/S1473-3099(26)00051-4)
Supplement: Supplementary appendix 4 [file mmc4.pdf]

THE LANCET
Infectious Diseases

Supplementary appendix 4

This appendix formed part of the original submission and has been peer reviewed. We post it as supplied by the authors.

Supplement to: Flores Girón L, Sganzerla Martinez G , Daniel BN, et al. Case presentation of patients hospitalised with mpox (subclade Ib/2023sh) including children, adolescents, and adults in South Kivu, Democratic Republic of the Congo: an observational cohort study. *Lancet Infect Dis* 2026; published online March 9. https://doi.org/10.1016/S1473-3099(26)00051-4.

Item	Page
Demographics and epidemiological details of all recruited participants	2-3
Measured outcomes	4
Statistical analysis plan	5
Molecular detection of MPXV subclade Ib/2023sh	6
DNA metagenomics sequencing	7
Phylogenetic analysis	8
Comparison of average number of reported symptoms per age group	9
Presence of symptoms in male/female patients of different age groups	10
Comparison of average number anatomical regions per age group	11
Presence of lesions in anatomical regions in male/female patients of different age groups	12
Sequencing results	13-14
Comparison of average oral swabs Ct values in different age groups according to presence/absence of skin lesions	15
Symptom profile of participants with and without skin lesions	16-17
Distribution of Ct values from skin swabs in different age groups	18
Prisma diagram of the literature search for age distribution of mpox cases in clinical cohorts	19
Inclusion criteria (in French)	20
References	21

Demographics and epidemiological details of all recruited participants

	Female	Male	Total
**Baseline characteristics**			
N	546/929 (58.8%, 95% CI 55.6-61.9)	383/929 (41.2%, 95% CI 38.1-44.4)	929/929
Median age [IQR] (95% CI])	14 [3-25] (95% CI 11-16)	4 [1-15.5] (95% CI 3 – 5)	8 [1.625-22] (95% CI 6-10)
< 1 year	85/546 (15.6%, 95% CI 12.8-18.8)	108/383 (28.2%, 95% CI 23.9-32.9)	193/929 (20.8%, 95% CI 18.3-23.5)
1-5 years	106/546 (19.4%, 95% CI 16.3-22.9)	108/383 (28.2%, 95% CI 23.9-32.9)	214/929 (23.0%, 95% CI 20.4-25.9)
6-10 years	57/546 (10.4%, 95% CI 8.1-13.3)	47/383 (12.3%, 95% CI 9.4-15.9)	104/929 (11.2%, 95% CI 9.3-13.4)
11-15 years	42/546 (7.7%, 95% CI 5.7-10.2)	24/383 (6.3%, 95% CI 4.2-9.2)	66/929 (7.1%, 95% CI 5.6-8.9)
16-20 years	66/546 (12.1%, 95% CI 9.6-15.1)	33/383 (8.6%, 95% CI 6.2-11.9)	99/929 (10.7%, 95% CI 8.8-12.8)
≥ 21 years	190/546 (34.8%, 95% CI 30.9-38.9)	63/383 (16.4%, 95% CI 13.1-20.5)	253/929 (27.2%, 95% CI 24.5-30.2)
Household size median [Q1-Q3] (95% CI)	6 [5-8] (95% CI 6-7)	6 [5-8] (95% CI 6-7)	7 [5-9] (95% CI 6-7)
People aged ≤ 15 in the same household median [Q1-Q3] (95% CI)	4 [3-6] (95% CI 3-4)	3 [2-6] (95% CI 2-4)	4 [3-6] (95% CI 3-4)
People aged >15 in the same household median [Q1-Q3] (95% CI)	2 [2-2] (95% CI 2-2)	2 [2-2] (95% CI 2-2)	2 [2-2] (95% CI 2-2)
**Risk factors**			
Contact with suspect mpox case n (%)	271/546 (49.6%, 95% CI 45.5-53.8)	135/383 (35.2%, 95% CI 30.6-40.2)	406/929 (43.7%, 95% CI 40.5-46.9)
Pregnancy n (%)	24/546 (4.4%, 95% CI 3.0-6.5)		
Active smoking n (%)	0/546 (0.0%, 95% CI 0.0-0.7)	4/383 (1.0%, 95% CI 0.4-2.7)	4/929 (0.4%, 95% CI 0.2-1.1)
Previous tuberculosis n (%)	0/546 (0.0%, 95% CI 0.0-0.7)	1/383 (0.3%, 95% CI 0.0-1.5)	1/929 (0.1%, 95% CI 0.0-0.6)
HIV n (%)	3/546 (0.5%, 95% CI 0.2-1.6)	3/383 (0.8%, 95% CI 0.3-2.3)	6/929 (0.6%, 95% CI 0.3-1.4)
Malaria rapid test, positive/total tested	23/192	19/150	42/216

Contact with domestic animals n (%)	37/546 (6.8%, 95% CI 5.0-9.2)	26/383 (6.8%, 95% CI 4.7-9.8)	63/929 (6.8%, 95% CI 5.3-8.6)
Contact with wild animals n (%)	3/546 (0.5%, 95% CI 0.2-1.6)	5/383 (1.3%, 95% CI 0.6-3.0)	8/929 (0.9%, 95% CI 0.4-1.7)
**Employment**			
Farmer, n (%)	141/546 (25.8%, 95% CI 22.3-29.7)	25/383 (6.5%, 95% CI 4.5-9.5)	166/929 (17.9%, 95% CI 15.5-20.5)
Student, n (%)	78/546 (14.3%, 95% CI 11.6-17.5)	58/383 (15.1%, 95% CI 11.9-19.1)	136/929 (14.6%, 95% CI 12.5-17.1)
Children below the local school age[1]	191/546 (35.0%, 95% CI 31.1-39.1)	216/383 (56.4%, 95% CI 51.4-61.3)	407/929 (43.8%, 95% CI 40.7-47.0)
Craftsman/merchant, n (%)	31/546 (5.7%, 95% CI 4.0-7.9)	12/383 (3.1%, 95% CI 1.8-5.4)	43/929 (4.6%, 95% CI 3.5-6.2)
Military, n (%)	2/546 (0.4%, 95% CI 0.1-1.3)	15/383 (3.9%, 95% CI 2.4-6.4)	17/929 (1.8%, 95% CI 1.1-2.9)
Public server, n (%)	1/546 (0.2%, 95% CI 0.0-1.0)	4/383 (1.0%, 95% CI 0.4-2.7)	5/929 (0.5%, 95% CI 0.2-1.3)
Unemployed, n (%)	54/546 (9.9%, 95% CI 7.7-12.7)	9/383 (2.3%, 95% CI 1.2-4.4)	63/929 (6.8%, 95% CI 5.3-8.6)

[1]The typical starting age for school in the study area is 6 years old.

Measured outcomes

Mpox lesions were tracked in the questionnaire in the format of different questions. First, one question regarding the estimate number of total body lesions was included (categorical; less than 10, between 11-50, between 51-100, more than 100, and more than 500). The number of lesions was estimated by hospital staff. In addition, individual yes/no questions were made if the patients had lesions in the following body parts: head, face, ears, neck, back, nose, nasal mucosa, forearm, arm, dorsal hand, palm of hand, chest, buttocks, front thighs, back thigs, legs, dorsal foot, plantar foot, and genital area. We created a new variable the tracks the number of 'YES' answers for each body part, representing the total number of body parts with mpox lesions reported. Patients had their mpox lesions palpitated and asked if they felt pain, if so, pain was classified into three levels by the hospital staff following a scale they created: *i*) Mild: the patient exhibits minimal reaction to gentle palpitation. Lesions are sensitive but not overtly painful. No vocalization of withdrawal observed. Patients allowed examination without resistance. Behavior and appetite are normal. Patients responded with a slight flinch or not reaction. *ii*) Moderate: the patient reacts noticeably to palpitation. Pain is evident upon touch but tolerable. The response might include withdrawal, vocalization, or tense posture. The patient may try to avoid contact but does not show extreme distress. Mild behavioral changes (e.g., reduced movement or appetite). Patients responded with a clear withdrawal or vocalization, but examination is possible. *iii*) Severe: the patient shows intense response to even minimal palpitation. Touch the lesion trigger immediate and strong reactions such as withdrawal, aggression, vocalization, or escape attempts. The patient may refuse to move, eat or be approached. Pain severely affects general condition. Patients responded strongly with screaming, biting, and rapid escape. Palpitation often not tolerated. The presence of the following symptoms was assessed by hospital staff during admission: fever, dysphagia/pharyngitis, cough, headache, lymphadenopathy, dyspnea, myalgia, weight loss, conjunctivitis, and vomiting.

Hospital staff assessed lymphadenopathy by palpitation. When palpitation revealed swelling of the lymph nodes either in the cervical or inguinal regions, lymphadenopathy was recorded as present. During the general examination, patients with conjunctivitis usually presented with eye discomfort, tearing, and irritation of the conjunctiva. In some cases, small lesions or wounds could even be observed on the conjunctival mucosa. It was common for conjunctivitis itself to cause discomfort and patients often spontaneously reported that their eyes did not feel right or felt irritated. Rapid malaria testing was also performed in consenting participants. A positive diagnosis of malaria was not an exclusion criterion.

Statistical analysis plan

Descriptive statistics will be reported as mean ± standard deviation for normally distributed continuous variables, median with interquartile range for non-normally distributed continuous variables, and frequency with percentage for categorical variables. Data normality will be ascertained using the Shapiro-Wilk test. Continuous variables will be compared using t-test, Mann-Whitney U test, ANOVA, or Kruskall-Wallis test, depending on number of groups and data distribution. Categorical variables will be compared using the chi-square test. Post-hoc analyses will be done on the comparison of continuous variables for three or more groups using Tukey's post hoc pairwise comparisons. Post hoc analysis of categorical variables will be done with an analysis of standardized residuals within the contingency table. Analysis of bimodal distribution is done using the Hartigan's Dip Test.

For categorical variables expressed as proportions, 95% confidence intervals (Cis) were calculated using the Wilson score method. For continuous variables, 95% Cis for the mean and medians were estimated using nonparametric bootstrap resampling (10,000 iterations) taking the 2.5^{th} and 97.5^{th} percentile of the bootstrap distribution as the confidence limits.

To explore the data, we used the Python (version 3.10) packages: pandas (version 2.2.3) for general data operations, scipy.stats (version 1.15.2), statsmodels (version 0.14.4), diptest (version 0.10.0), and numpy (version 2.2.5) for statistical analyses and mathematical operations, and matplotlib (version 3.10.1) and seaborn (version 0.13.2) for plotting and visuals. A p-value of <0.05 will be considered statistically significant. A residual value of ≥|1.96| will be considered statistically significant at the 0.05 level.

Molecular diagnostics of mpox virus

To detect MPXV subclade Ib, we employed an allele-specific real-time quantitative PCR (ARMS-qPCR) assay adapted to a Fast 7500 Applied Biosystems or a Bio-Rad CFX Opus 96 platform, using the Luna® Universal Probe qPCR Master Mix (New England Biolabs). This assay is designed as a singleplex reaction targeting a subclade Ib–specific single-nucleotide polymorphism region, providing high sensitivity and specificity for lineage-level discrimination. Custom oligonucleotides were synthesized by Integrated DNA Technologies (IDT). The subclade Ib forward primer sequence is 5'-GGA TGT GGA CAT TTA ACA ATC TG-3', the reverse primer is 5'-CTT CCA AAC TTA ATC ACT CCT AG-3', and the hydrolysis probe is 5'-/Cy5/TC AGG CGC ATA TCC ACC CAC GT/3BHQ_2/-3' as proposed and validated by Xu et al (2025). Stocks are be reconstituted to 100 μM in nuclease-free water and stored at −20 °C, with 10 μM working dilutions prepared for routine use. Each 20 μL reaction contains 10.0 μL Luna Universal Probe qPCR Master Mix (2×), 0.4 μL forward primer (10 μM; final 0.2 μM), 0.4 μL reverse primer (10 μM; final 0.2 μM), 0.2 μL probe (10 μM; final 0.1 μM), 4.0 μL nuclease-free water, and 5.0 μL containing 2.5 ng of DNAof extracted DNA template. Master mixes are prepared on ice with a 10% excess volume to ensure pipetting accuracy. Reactions were run under the following cycling conditions: 95 °C for 5 min (initial denaturation), followed by 40 cycles of 95 °C for 10 s and 60 °C for 30 s, with fluorescence acquired in the Cy5 channel at the annealing/extension step. Each run includes a positive control (validated MPXV subclade Ib DNA or synthetic fragment) and a no-template control (NTC) to monitor for contamination. A sample is considered positive for subclade Ib if amplification is observed with a Cq < 40 and a characteristic sigmoidal curve. Samples with no amplification or Cq ≥ 40 are reported as negative by this assay.

DNA metagenomics sequencing

Sequencing was performed at CSRN-Lwiro or IWK Health Center by utilizing the Oxford Nanopore Rapid Sequencing DNA - PCR Barcoding Kit 24 V14. High molecular weight genomic DNA, ranging from 1-5 ng, was extracted and quantified, and its purity and integrity verified to ensure successful library preparation. The DNA was tagmented using the Fragmentation Mix provided in the kit. Barcoded primers from the kit are added for PCR amplification. Sequencing adapters were attached to the PCR products. The PCR includes an initial denaturation at 95°C for three minutes, which is followed by cycles of denaturation at 95°C for 15 seconds, annealing at 56°C for 15 seconds, and extension at 65°C for six minutes. A final extension was conducted at 65°C for six minutes. After PCR amplification, the products were cleaned using AMPure XP magnetic beads and quantified using the Qubit fluorometer to ensure the correct concentration for sequencing. The library was prepared for sequencing and loaded onto an R10.4.1 flow cell after priming, using the MinION system. The library was loaded via the SpotON sample port. Sequencing was conducted over 24 hours, with real-time monitoring and data quality assessment enabled through the ONT MinKNOW software. Base calling was executed using MinKNOW with a minimum quality score (Q score) of 9. The generated reads in FASTQ format are then submitted to the fastp tool for quality control, quality filtering, and adapter trimming. Filtered reads are aligned using Minimap2 (version 2.26r1175) to the MPXV Clade I reference genome (GenBank accession NC_003310.1). The alignment BAM files were sorted, indexed, and used to generate a consensus sequence with samtools (version 1.19.2).

Phylogenetic analysis

A total of 536 MPXV Clade Ib genomes were downloaded from the Global Initiative on Sharing All Influenza Data (GISAID) (https://gisaid.org/) (Khare et al., 2021) in the fasta format. These genome sequences were submitted from as many as 16 countries and territories between 2023-10-05 to 2025-03-29 (EPI_SET_250524qc). In addition to these Clade Ib genomes, we have also downloaded a total of 39 Clade Ia genomes from GenBank (Sayers et al., 2024). To calculate a combined tree of reported Clade Ia and Ib and two Lwiro sequences, initially, a multiple sequence alignment was performed with the aid of the Minimap2 v2.17 tool (Li et al., 2018) embedded in the Squirrel pipeline (https://github.com/aineniamh/squirrel). In the next step, masking and quality control were performed, considering the NC_003310.1 genome as the default reference. Two genomes (KJ642617 and KJ642615) were considered as outgroup sequences. After obtaining the combined MSA of reported Clade Ia, b, and Lwiro sequences, the best substitution model was selected using the Modelfinder program integrated with the IQ-TREE package (Minh et al., 2020). Finally, a Maximum-likelihood phylogeny tree containing Clade Ia, b, and Lwiro sequences was computed using the IQ-TREE multicore package version 2.2.6 (Minh et al., 2020) with 1,000 bootstrap iterations.

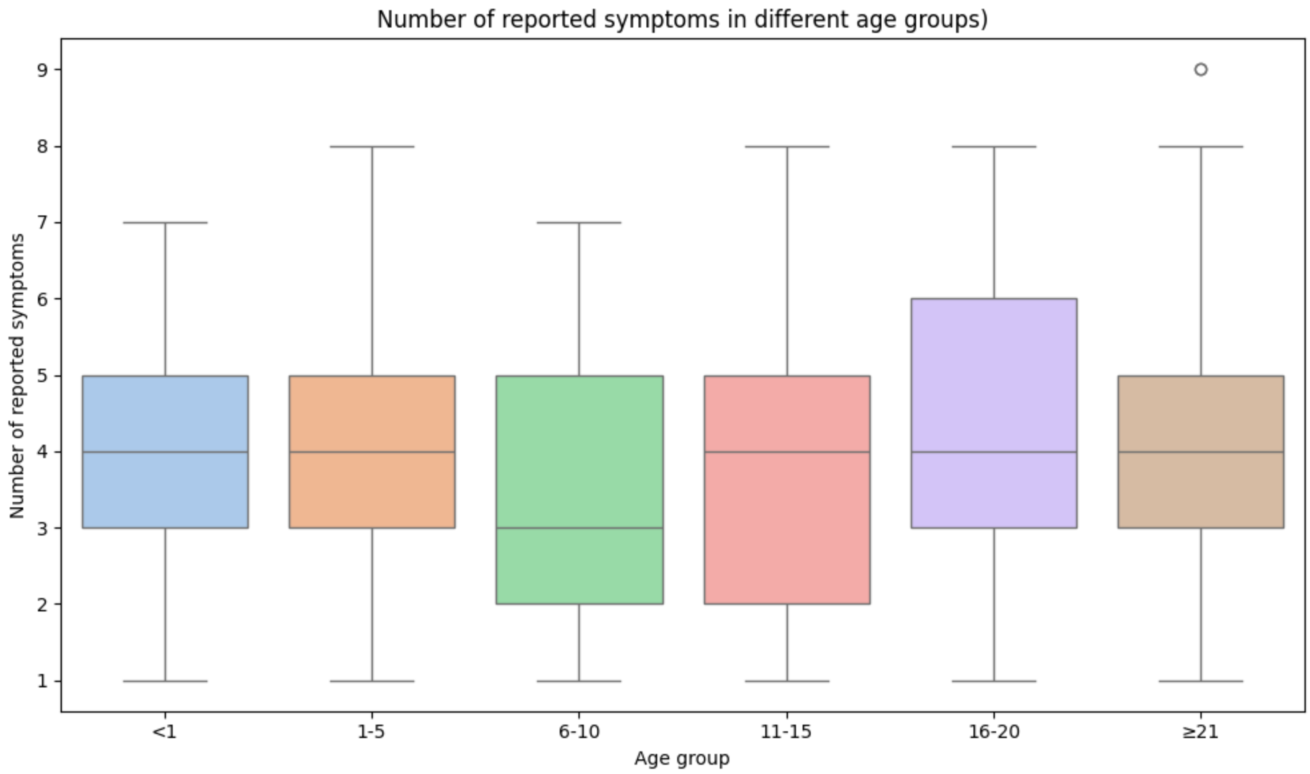

The average number of symptoms reported per study participant (non-parametric, Shapiro-Wilk *p*-value < 0.001) was 3.93±1.64, median = 4, Q1-Q3 = 3-5, 95% CI = 4-4. No statistical significance (Kruskal-Wallis *p*-value = 0.40) was found in comparing the average number of reported symptoms in each age group.

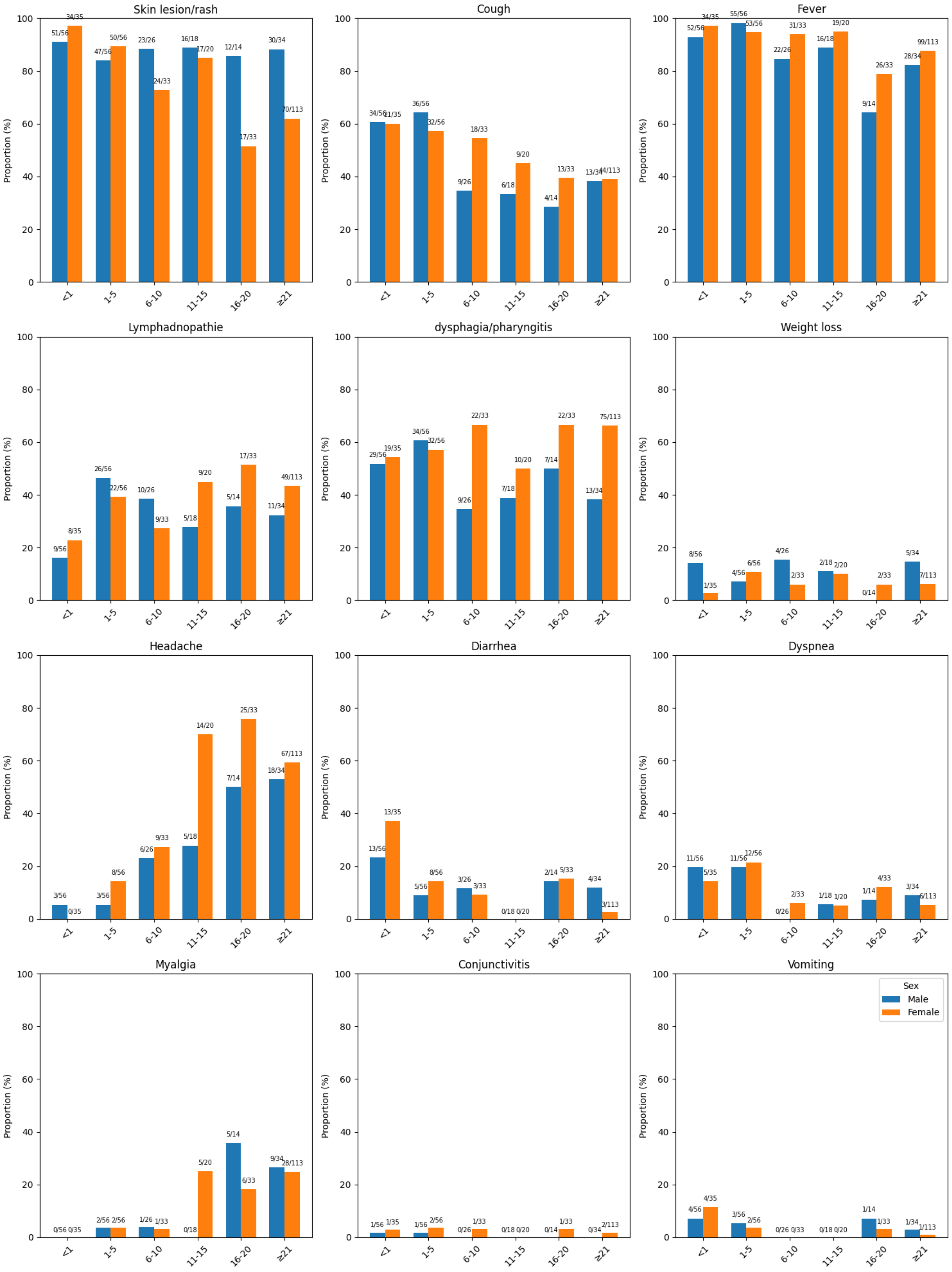
Skin lesion/rash
Cough
Fever
Lymphadnopathie
dysphagia/pharyngitis
Weight loss
Headache
Diarrhea
Dyspnea
Myalgia
Conjunctivitis
Vomiting
Proportion (%)
0
20
40
60
80
100
<1
1-5
6-10
11-15
16-20
≥21
Sex
Male
Female
51/56 34/35 47/56 50/56 23/26 24/33 16/18 17/20 12/14 17/33 30/34 70/113
34/56 21/35 36/56 32/56 9/26 18/33 6/18 9/20 4/14 13/33 13/34 44/113
52/56 34/35 55/56 53/56 22/26 31/33 16/18 19/20 9/14 26/33 28/34 99/113
9/56 8/35 26/56 22/56 10/26 9/33 5/18 9/20 5/14 17/33 11/34 49/113
29/56 19/35 34/56 32/56 9/26 22/33 7/18 10/20 7/14 22/33 13/34 75/113
8/56 1/35 4/56 6/56 4/26 2/33 2/18 2/20 0/14 2/33 5/34 7/113
3/56 0/35 3/56 8/56 6/26 9/33 5/18 14/20 7/14 25/33 18/34 67/113
13/56 13/35 5/56 8/56 3/26 3/33 0/18 0/20 2/14 5/33 4/34 3/113
11/56 5/35 11/56 12/56 0/26 2/33 1/18 1/20 1/14 4/33 3/34 6/113
0/56 0/35 2/56 2/56 1/26 1/33 0/18 5/20 5/14 6/33 9/34 28/113
1/56 1/35 1/56 2/56 0/26 1/33 0/18 0/20 0/14 1/33 0/34 2/113
4/56 4/35 3/56 2/56 0/26 0/33 0/18 0/20 1/14 1/33 1/34 1/113

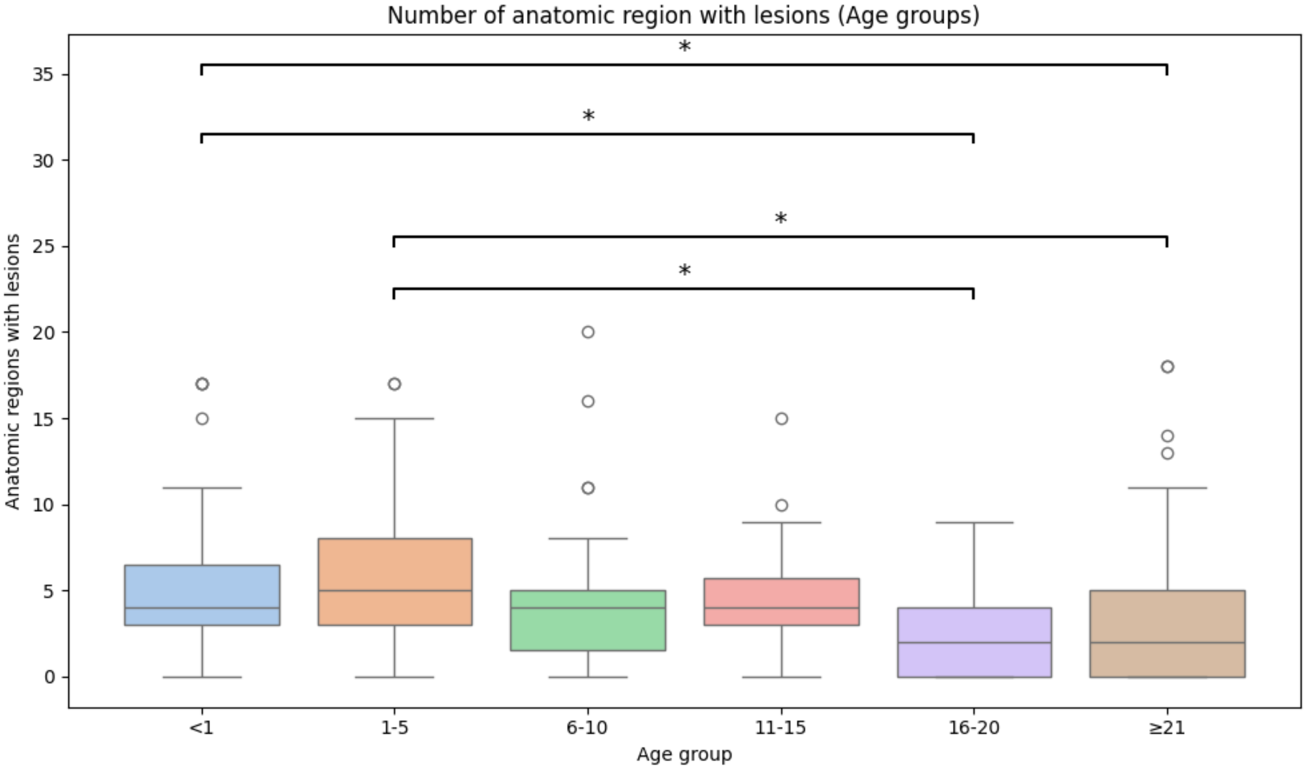

The average number of symptoms reported per study participant (non-parametric, Shapiro-Wilk *p*-value < 0.001) was 4.2±3.7, median = 4, Q1-Q3 = 2-6, 95% CI = 3-4. A one-way ANOVA yielded significant (p-value < 0.001) between the groups. Tukey post-hoc pairwise comparisons revealed significantly higher mean in the age groups i) <1 vs 16-20 (p-value <0.0001), ii) <1 vs ≥21 (p-value <0.0001), 1-5 vs 16-20 (p-value < 0.001), and 1-5 vs ≥21 (p-value < 0.001). In conclusion, the average number of lesions was generally higher in younger study participants.

Age group	Mean± Standard deviation	Median [IQR]	95% CI
<1	5.09±3.66	4 [3-4]	4-5
1-5	5.57±4.07	5 [3-8]	4-6
6-10	4.06±3.78	4 [1.5-5]	3-4
11-15	4.44±3.14	4 [3-5.75]	3-5
16-20	2.57±2.45	2 [0-4]	1-3
≥21	3.11±3.38	2 [0-5]	2-3

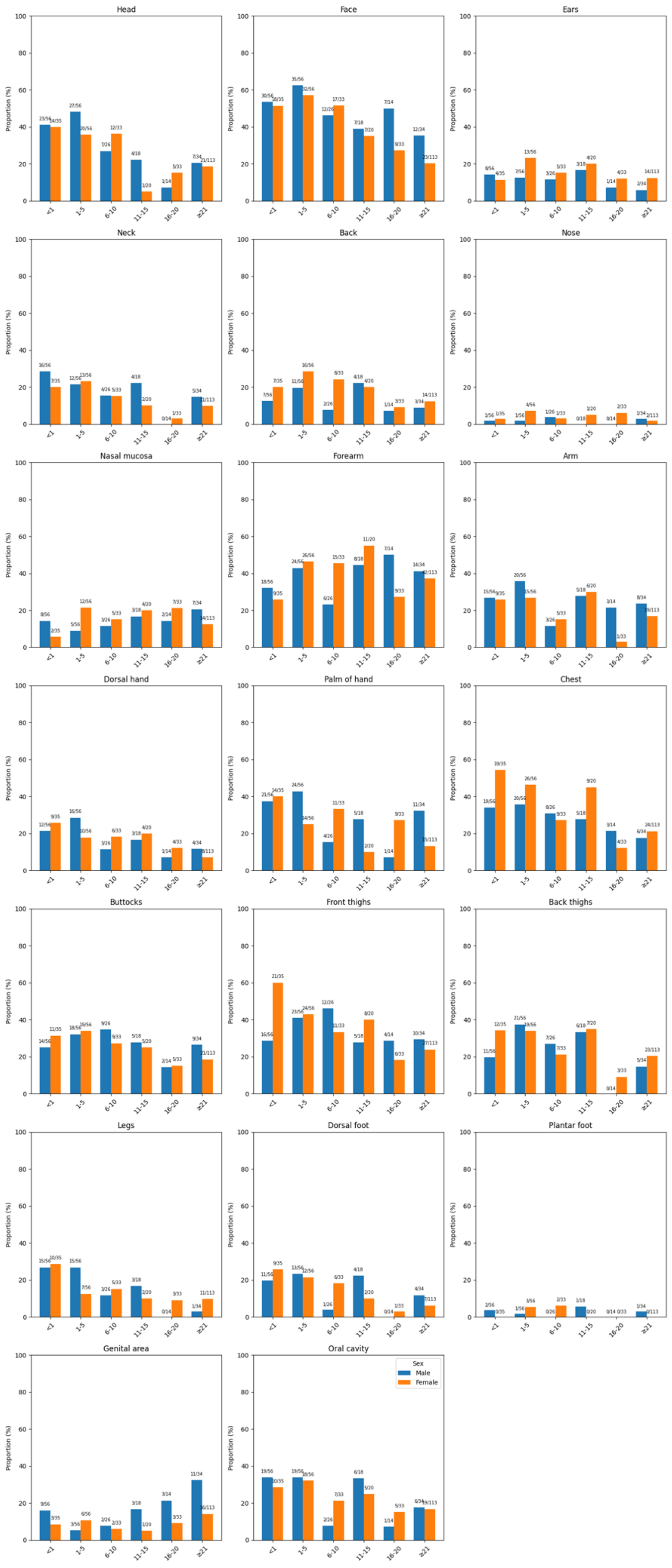
Head
Face
Ears
Neck
Back
Nose
Nasal mucosa
Forearm
Arm
Dorsal hand
Palm of hand
Chest
Buttocks
Front thighs
Back thighs
Legs
Dorsal foot
Plantar foot
Genital area
Oral cavity
Proportion (%)
<1
1-5
6-10
11-15
16-20
≥21
Sex
Male
Female

DNA metagenomic sequencing results.

GISAID	Collection date	Swab	Length of consensus (bp)	Percentage of N (%)	Total reads (k)	Aligned reads	Coverage (x)	Average read length
hMPXV-DRC-CRSN-0010-2024	2024-12-03	Skin	195,428	0.36	1,307.215	245,042	4,833.79	3,883.3
hMPXV-DRC-CRSN-0200-2024	2024-12-03	Skin	195,538	0.32	1,931.889	2,988	63.91	4,211.1
hMPXV-DRC-CRSN-0100-2024	2024-10-02	Skin	195,424	0.006	263,491	21,811	353.63	3,191.8
hMPXV-DRC-CRSN-353-2024	2024-09-22	Skin	195,373	0.16	360,323	2,179	37.66	3,407.2
hMPXV-DRC-CRSN-20-2024	2024-10-02	Skin	195,596	1.25	471,155	743	14.36	3,807.1

hMPXV-DRC-CRSN-83-2024	2024-10-25	Oral	195,481	3.22	233.593	221	4.51	4,090.7
hMPXV-DRC-CRSN-215-2024	2024-09-18	Oral	195,482	0.005	271.256	37,688	556.96	4,022.7
hMPXV-DRC-CRSN-211-2024	2024-09-12	Oral	195,498	0.10	95.063	2,198	45.28	4,056.2
hMPXV-DRC-CRSN-204-2024	2024-09-22	Oral	195,583	5.62	17.529	258	4.86	3,771.4
hMPXV-DRC-CRSN-171-2024	2024-10-09	Oral	193,877	2.43	845.979	545	11.75	4,247

Comparison of average oral swabs Ct values in different age groups according to presence/absence of skin lesions.

Age group	Lesion			No lesion			Median comparison (Mann Whitney U test p-value)
	N	Median, IQR, 95% CI	Modality	N	Median, IQR, 95% CI	Modality	
All ages	317	30.40, 24.27-37.08, 29.18-32.11	Unimodal, p<0.05	93	32.41, 26.24-37.51, 30.32-34.69	Multimodal, p=0.61	0.27
<1	74	31.61, 25.90-36.67, 29.19-34.37	Multimodal, p=0.75	5	34.69, 22.66-37.32, 22.61-38.48	Unimodal, p<0.05	0.96
1-5	83	26.46, 22.75-37.20, 24.02-30.40	Unimodal, p<0.05	14	34.53, 28.05-37.70, 29.81-37.76	Multimodal, p=0.32	0.12
6-10	35	35.26, 26.59-38.38, 28.90-37.29	Multimodal, p=0.37	11	32.41, 28.84-38.13, 27.57-38.33	Multimodal, p=0.23	0.72
11-15	22	33.83, 29.25-37.52, 29.48-37.34	Multimodal, p=0.76	5	25.98, 22.55-36.02, 22.32-39.79	Multimodal, p=0.31	0.71
16-20	21	31.19, 24.68-36.63, 26.08-34.24	Multimodal, p=0.94	17	33.55, 25.47-38.30, 25.47-38.30	Multimodal, p=0.71	0.75
≥21	82	29.95, 24.80-36.14, 27.53-32.57	Multimodal, p=0.26	41	30.87, 27.26-35.42, 28.02-34.57	Multimodal, p=0.40	0.55

Symptom profile of participants with and without skin lesions

Comparison of symptom frequency in participants with and without skin lesions across different age groups. The heigh of each bar represents the proportion of participants in each category: participants with skin lesion (n=282) and without skin lesions (n=113) who tested positive by PCR in skin or oral cavity/oropharynx samples. Each bar is annotated with the absolute counts of participants exhibiting the symptom and the corresponding Wilson 95% confidence interval of the proportion. Chi-square tests were performed for each symptom between groups, and only significant p-values are shown above the bars. Significant differences in symptoms between groups (with residual ≥1.96) are visually reflected in the relative heights of the bars.

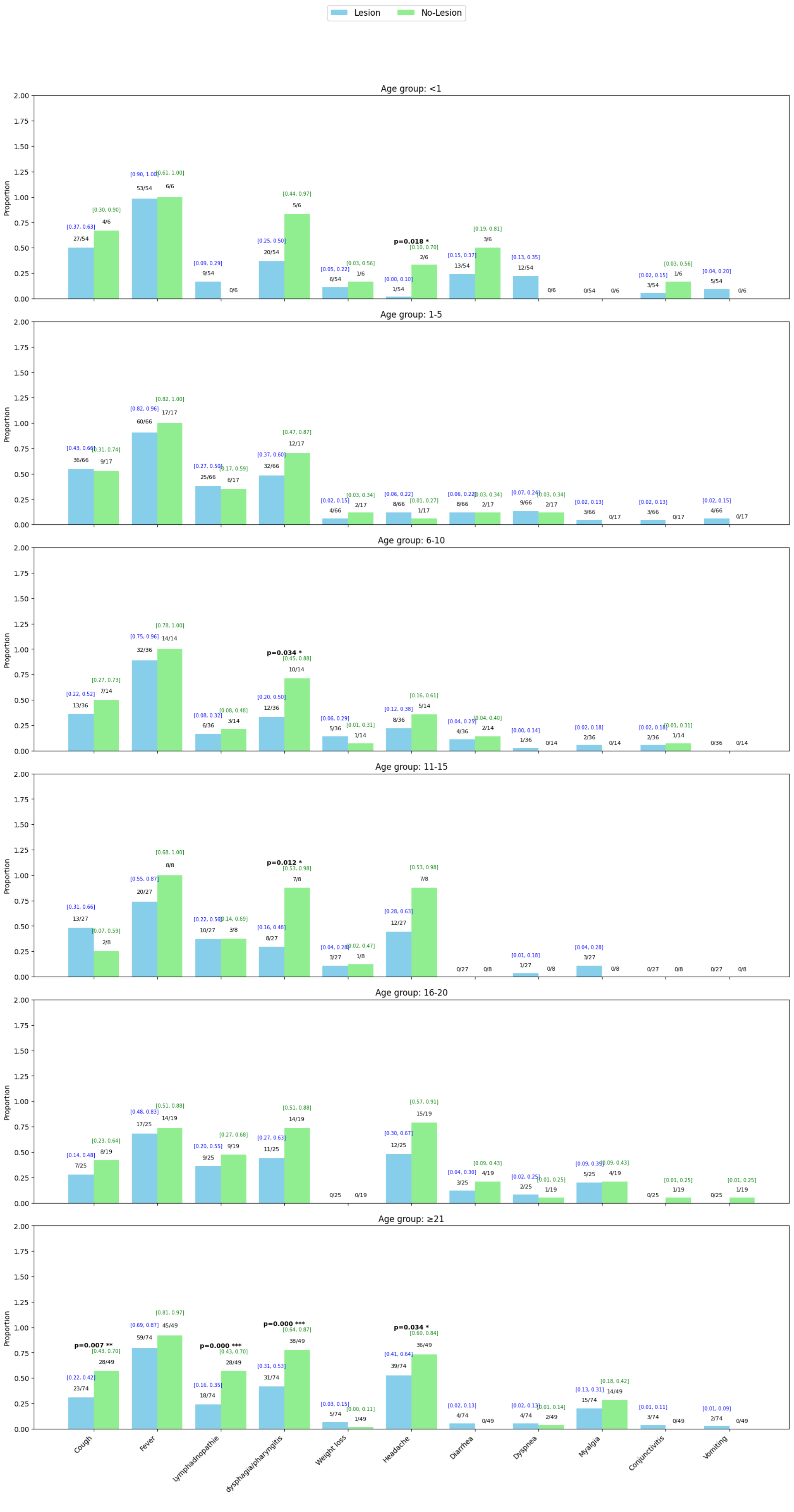
Lesion
No-Lesion
Age group: <1
p=0.018 *
Age group: 1-5
Age group: 6-10
p=0.034 *
Age group: 11-15
p=0.012 *
Age group: 16-20
Age group: ≥21
p=0.007 **
p=0.000 ***
p=0.000 ***
p=0.034 *
Proportion
0.00
0.25
0.50
0.75
1.00
1.25
1.50
1.75
2.00
Cough
Fever
Lymphadnopathie
dysphagia/pharyngitis
Weight loss
Headache
Diarrhea
Dyspnea
Myalgia
Conjunctivitis
Vomiting

Distribution of Ct values from skin swabs in different age groups

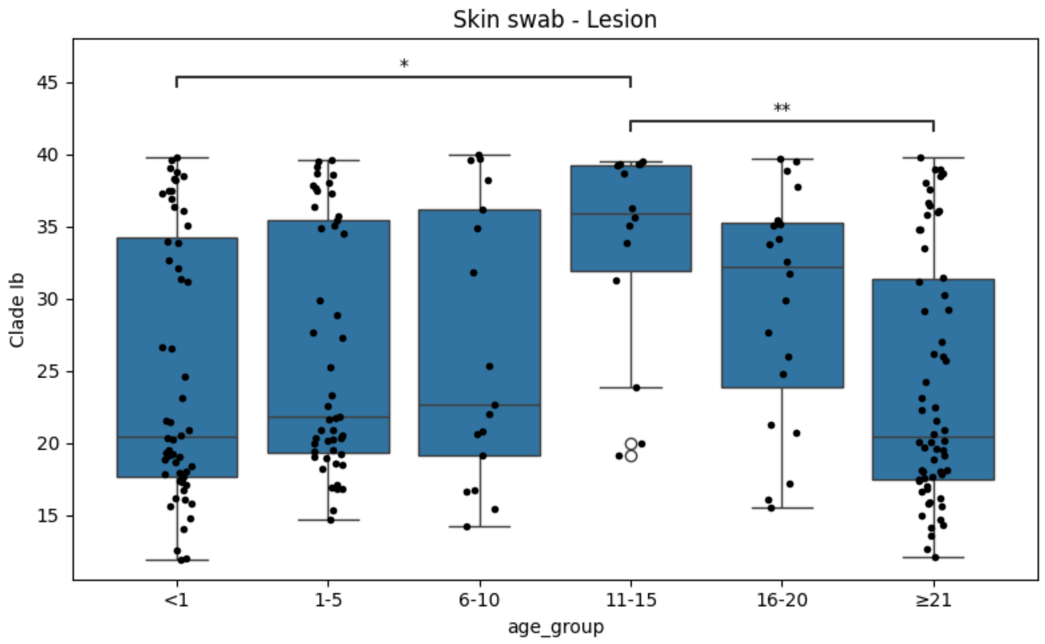

We compared the median Ct value of skin swabs from different age groups. Statistically significant differences were found in at least two groups (Kruskal-Wallis p-value<0.001). Dunn posthoc pairwise comparison revealed significant difference in the medians of the age groups <1 (n=56, median 20.42, IQR=17.68-34.25, 95% CI 18.95-26.67) vs 11-15 (n=14, median 35.94, IQR=31.91-39.29, 95% CI 31.24-39.31), and 11-15 vs 21≥ (n=62, median 20.41, IQR=17.47-31.40, 95% CI 18.65-25.69) with both pairwise comparisons returning p-values <0.05.

Prisma diagram of the literature search for age distribution of mpox cases in clinical cohorts

We searched PubMed on Sep 26, 2025, for cohort studies using the terms (“monkeypox”, OR “mpox”, OR “monkey pox”, OR “monkeypox virus”, OR “MPXV”, OR “monkey pox virus”) to find information on age distribution of cases. Initially we identified 173 articles of which 48 were included for full text analysis. Out of 48 eligible articles, 8 were excluded as they had insufficient demographic details to ascertain pediatric cases. In total, 40 articles were included.

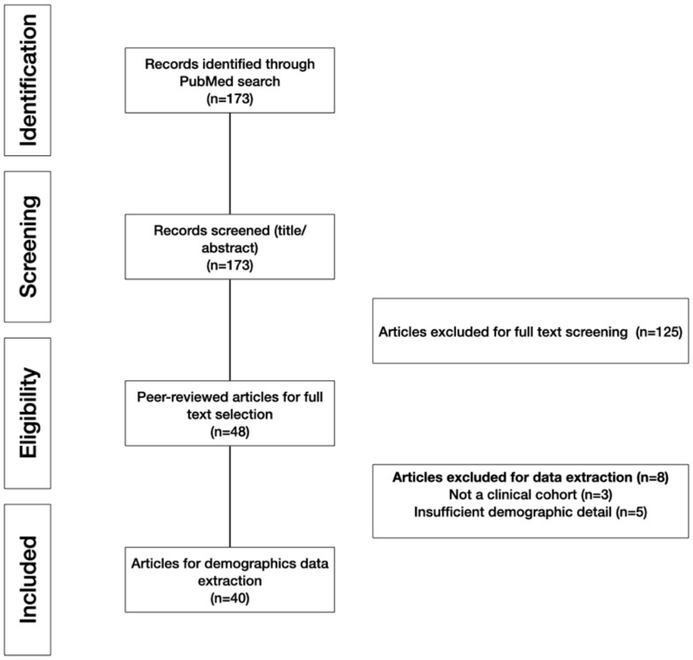

Inclusion criteria

Les critères d'inclusion utilisés comprenaient les individus de tout âge résidant dans la zone de santé de Miti-Murhesa, où l'épidémie était endémique et présentait une forte transmission intracommunautaire. Les participants devaient présenter, au moment de l'inclusion, des lésions cutanées compatibles avec l'infection. Étaient également éligibles les individus ne présentant pas de lésions compatibles avec une infection par le mpox, mais manifestant au moins l'un des symptômes suivants : fièvre, adénopathie cervicale ou pharyngite, à condition d'avoir été en contact, au cours des 21 jours précédents, avec un cas suspect de mpox. Toutes les personnes incluses dans l'étude ont été soumises à un test rapide de dépistage du paludisme, qui est également endémique dans la zone d'étude au moment de l'inclusion. Les critères d'inclusion adoptés dans notre recherche étaient basés sur les recommandations de la zone de santé de Miti-Murhesa, dans la province du Sud-Kivu. L'inclusion des participants était également définie par le jugement des cliniciens.

REFERENCES

Li, H. (2018). Minimap2: Pairwise alignment for nucleotide sequences. Bioinformatics, 34(18). https://doi.org/10.1093/bioinformatics/bty191

Minh, B. Q., Schmidt, H. A., Chernomor, O., Schrempf, D., Woodhams, M. D., von Haeseler, A., Lanfear, R., & Teeling, E. (2020). IQ-TREE 2: New Models and Efficient Methods for Phylogenetic Inference in the Genomic Era. *Molecular Biology and Evolution*, *37*(5). https://doi.org/10.1093/molbev/msaa015

Shruti Khare, Céline Gurry, Lucas Freitas, Mark B Schultz, Gunter Bach, Amadou Diallo, Nancy Akite, Joses Ho, Raphael TC Lee, Winston Yeo, GISAID Core Curation Team, Sebastian Maurer-Stroh. GISAID's Role in Pandemic Response[J]. *China CDC Weekly*, 2021, 3(49): 1049-1051. doi: 10.46234/ccdcw2021.255

Sayers, E. W., Cavanaugh, M., Clark, K., Pruitt, K. D., Sherry, S. T., Yankie, L., & Karsch-Mizrachi, I. (2024). GenBank 2024 Update. *Nucleic Acids Research*, *52*(D1). https://doi.org/10.1093/nar/gkad903

Xu, B., Xiong, D., Zhang, X., Wei, H., & Yu, J. (2025). Development of an ARMS-Quadruplex-qPCR assay for the rapid identification of MPXV and the clades Ia, Ib, IIa and IIb. *Journal of Virological Methods*, *334*. https://doi.org/10.1016/j.jviromet.2025.115125
